# Fraxetin Inhibits UGT1A1 and UGT1A9 Activities In Vitro: Inhibition Kinetics, Molecular Dynamics Simulation, and Prediction of Herb–Drug Interaction Risk

**DOI:** 10.3390/ph19060968

**Published:** 2026-06-22

**Authors:** Jinqian Chen, Han Han, Jibin Li, Simeng Xu, Xichuan Li, Zhenyu Zhao

**Affiliations:** 1 NHC Key Laboratory of Hormones and Development, Tianjin Key Laboratory of Metabolic Diseases, Chu Hsien-I Memorial Hospital & Institute of Endocrinology, Tianjin Medical University, Tianjin 300134, China; chenjinqian07@163.com (J.C.); 13602019858@163.com (J.L.); 15022465673@163.com (S.X.); 2 School of Pharmacy, Nankai University, Tianjin 300350, China; hanhan184622@163.com; 3 Tianjin Key Laboratory of Animal and Plant Resistance, College of Life Sciences, Tianjin Normal University, Tianjin 300387, China

**Keywords:** fraxetin, Cortex Fraxini, UDP-glucuronosyltransferases, herb–drug interactions, inhibition kinetics, mixed-type inhibition, molecular dynamics simulation, IVIVE

## Abstract

**Background/Objectives:** Fraxetin (7,8-dihydroxy-6-methoxycoumarin), a coumarin constituent of Cortex Fraxini (Qinpi) used in traditional Chinese medicine, is metabolised mainly by UGT1A9, but its potential to inhibit UGT enzymes and cause herb–drug interactions (HDIs) is largely unstudied. **Methods:** Fraxetin and four related coumarins were screened against 11 recombinant human UGTs; isoforms inhibited ≥80% underwent full kinetic analysis with 4-methylumbelliferone as probe. Binding was examined by molecular docking on AlphaFold structures with PLIP, triplicate 100 ns molecular dynamics, and MM/GBSA and MM/PBSA free-energy calculations, and interaction risk by FDA 2020 in vitro–in vivo extrapolation (IVIVE). **Results:** Fraxetin alone inhibited both UGT1A1 and UGT1A9 by >80% and was characterised in detail, acting as a mainly competitive mixed-type inhibitor (UGT1A1 IC_50_ 15.99 μM, *K*_i_ 8.32 μM; UGT1A9 IC_50_ 8.44 μM, *K*_i_ 5.90 μM). A structure–activity comparison identified a dual-element pharmacophore comprising the C-6 methoxy group and the 7,8-dihydroxycoumarin aglycone. MM/GBSA favoured UGT1A9 over UGT1A1 (ΔΔ*G* = −4.06 kcal/mol, *p* = 0.005), concordant with the kinetic ranking. IVIVE predicted a borderline systemic signal (*R*_1_ > 1.02) but an intestinal *R*_1,gut_ approximately five- to seven-fold above the high-risk threshold of 11 after capping the luminal concentration at fraxetin aqueous solubility. **Conclusions:** This is the first characterisation of fraxetin as a moderate-potency inhibitor of UGT1A1 and UGT1A9 and points to a previously under-recognised herb–drug interaction risk concentrated in the intestinal lumen rather than systemically; the finding constitutes an interaction signal requiring clinical confirmation rather than an established risk.

## 1. Introduction

UDP-glucuronosyltransferases (UGTs) constitute a superfamily of phase II drug-metabolising enzymes that catalyse the glucuronidation of approximately 40–70% of clinically used drugs and numerous herbal constituents, converting lipophilic compounds into water-soluble glucuronides amenable to renal or biliary excretion [1,2]. Among the major hepatic and intestinal UGT isoforms—including UGT1A1, UGT1A3, UGT1A4, UGT1A6, UGT1A9, UGT2B7, and UGT2B15—UGT1A1 and UGT1A9 are of particular clinical importance given the breadth and medical significance of their substrate portfolios [1,3]. Recognition of the clinical relevance of UGT-mediated drug interactions has prompted both the U.S. Food and Drug Administration (FDA) and the European Medicines Agency (EMA) to recommend systematic evaluation of UGT inhibition potential for new drugs and herbal constituents prior to clinical use [4]. Among herbal constituents, those that are themselves UGT substrates pose particular concern as potential inhibitors.

*Cortex Fraxini* (Qinpi) is a commonly used TCM listed in the 2020 edition of the Chinese Pharmacopoeia [5]. With a clinical history spanning over 2000 years, it is prescribed for heat-clearing, dampness-drying, dysentery relief, and vision improvement at typical daily doses of 6–12 g [6,7]. Modern pharmacological investigations have revealed that the principal active coumarins of Cortex Fraxini—esculetin, esculin, fraxin, and fraxetin—possess diverse biological activities, including anti-inflammatory, antioxidant, antidiabetic, and anticancer properties [8,9]. Fraxetin (7,8-dihydroxy-6-methoxycoumarin), the aglycone form, is generated in vivo by intestinal microflora hydrolysis of its glucoside precursor fraxin following oral administration of Cortex Fraxini preparations, making it the predominant bioavailable form [10]. Subsequent systemic absorption leads to extensive phase II metabolism: UGT1A9 has been identified as the major enzyme responsible for fraxetin-7-O-glucuronidation and fraxetin-8-O-glucuronidation in human liver microsomes, with intrinsic clearance values of 42–300 μL/min/mg protein [11,12]. This extensive intestinal and hepatic first-pass glucuronidation results in limited oral bioavailability in rats [11]. The consequent accumulation of unabsorbed fraxetin in the intestinal lumen generates high local concentrations with direct implications for herb-drug interaction risk.

Accumulating evidence shows that natural compounds acting as UGT substrates can also inhibit the same or related UGT isoforms—a dual substrate-inhibitor property that may lead to significant herb-drug interactions (HDIs) by competitively displacing co-administered drugs from their metabolic pathways [13]. This phenomenon has been observed with various herbal constituents: for example, silybin from milk thistle strongly inhibits UGT1A1-mediated SN-38 glucuronidation, with intestinal inhibition risk ratios (*R*_1,gut_) exceeding 11 across all dosing scenarios tested, indicating a potentially significant intestinal HDI risk when combined with irinotecan [14]. Despite the documented traditional and clinical use of Cortex Fraxini-containing preparations, the inhibitory potential of its major coumarin constituents toward UGT isoforms has never been systematically evaluated. Among these constituents, fraxetin represents the highest-priority candidate given its established identity as a UGT1A9 substrate and its status as the predominant circulating form following oral administration—properties that make it the highest-priority candidate for investigation as a potential dual substrate-inhibitor. This knowledge gap carries direct patient safety implications that the present study aims to address.

The present study is framed as prospective hazard identification rather than as a response to documented clinical events: to our knowledge and based on a search of PubMed and the published pharmacovigilance literature, no clinical herb–drug interaction involving Cortex Fraxini or fraxetin and a UGT substrate drug has been reported to date. This absence is not, in itself, evidence of safety. Systematic reviews of herbal pharmacovigilance have repeatedly shown that herb–drug interactions are substantially under-reported, because herbal use is frequently not elicited during history-taking, patients often do not disclose herbal consumption, and post hoc causal attribution to a herbal product is rarely conclusive [15,16,17]. For Cortex Fraxini, whose clinical use is concentrated in East Asia, the geographic reach of spontaneous reporting systems such as FDA FAERS and EudraVigilance is further limited. Proactive in vitro and in silico characterisation of UGT inhibitory potential is therefore warranted before clinical signals emerge, particularly for a Pharmacopoeia-listed traditional medicine with a large user population.

The objective of this study was to fill this gap using a multi-method integrated approach: (1) characterising the inhibitory effects of fraxetin and its main structural analogues in Cortex Fraxini on clinically relevant UGT isoforms, including IC_50_ determination and comprehensive inhibition kinetic analysis to establish inhibition constants (*K*_i_) and mechanisms for the primary targets; (2) utilising molecular docking, protein-ligand interaction profiling (PLIP), and three independent 100 ns molecular dynamics (MD) replicates with MM/GBSA and MM/PBSA binding free energy calculations to gain structural and dynamic insights into isoform selectivity; and (3) applying the FDA 2020 static IVIVE model to quantitatively assess hepatic and intestinal HDI risk.

## 2. Results

### 2.1. Inhibitory Effects of Fraxetin and Its Structural Analogues on UGT Isoforms

**Figure 1:**
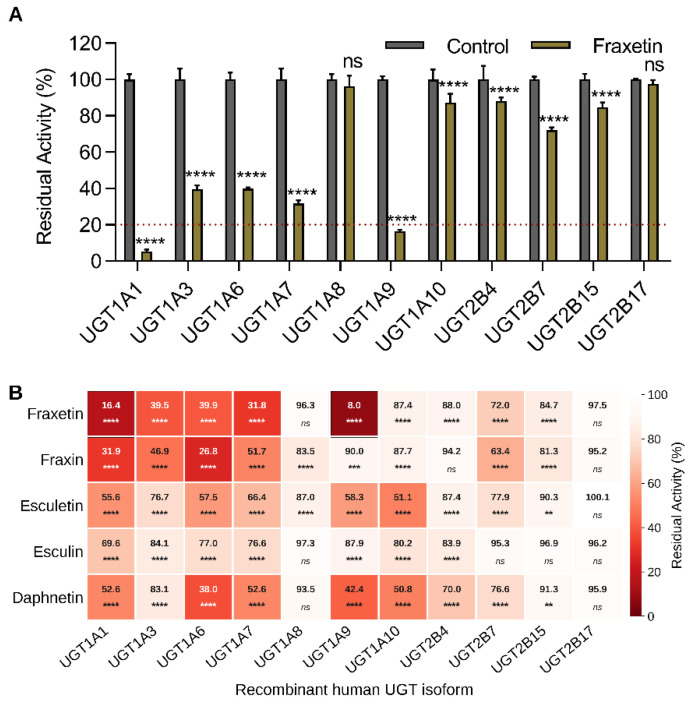
Inhibitory effects of the five Cortex Fraxini coumarins on 11 human recombinant UGT isoforms at a screening concentration of 100 μM (mean ± SE, *n* = 3). (**A**) Residual activity of fraxetin versus the vehicle control across the panel; the red dotted line marks 20% residual activity (≥80% inhibition), the threshold for kinetic follow-up. (**B**) Heat map of residual activity for all five coumarins (fraxetin, fraxin, esculetin, esculin and daphnetin); the colour scale indicates residual activity (%) and black outlines mark residual activity ≤ 20%. Residual activity was calculated relative to the solvent control. Statistical significance versus the vehicle control was assessed by two-way ANOVA (isoform × compound) followed by Dunnett’s multiple-comparison test within each isoform (**** *p* < 0.0001, *** *p* < 0.001, ** *p* < 0.01; ns, not significant); complete results are provided in Table S1.

The inhibitory effects of four major coumarin constituents of Cortex Fraxini (fraxetin, fraxin, esculetin, and esculin) and one structural analogue (daphnetin) were evaluated against 11 recombinant human UGT isoforms at 100 μM (Figure 1). Among the five compounds tested, fraxetin demonstrated the most pronounced and isoform-selective inhibitory profile, strongly inhibiting UGT1A1 and UGT1A9 with residual activities of 16.5 ± 2.3% and 8.0 ± 0.7%, respectively, corresponding to inhibition exceeding 80% for both isoforms. Moderate inhibition by fraxetin was additionally observed toward UGT1A3 (residual activity 39.5 ± 2.4%), UGT1A6 (39.9 ± 0.7%), and UGT1A7 (31.8 ± 1.6%), while minimal inhibitory effects were detected against UGT1A8, UGT1A10, UGT2B4, UGT2B7, UGT2B15, and UGT2B17 (residual activity > 70%). Fraxetin was the only compound to exceed 80% inhibition simultaneously for both UGT1A1 and UGT1A9 and was therefore selected for subsequent kinetic characterisation against these two isoforms. Two-way ANOVA (isoform × compound) revealed significant main effects of compound (F(5132) = 683.0, *p* < 0.0001) and isoform (F(10,132) = 402.4, *p* < 0.0001) and a significant interaction (F(50,132) = 72.26, *p* < 0.0001), confirming that the inhibitory profile was both compound- and isoform-dependent. By Dunnett’s multiple-comparison test against the vehicle control within each isoform, fraxetin significantly inhibited both UGT1A1 and UGT1A9 (both *p* < 0.0001), whereas it did not significantly affect UGT1A8 or UGT2B17 (*p* > 0.05); the complete per-compound, per-isoform comparisons are summarised in Table S1 and annotated on Figure 1.

The remaining compounds exhibited isoform-selective or moderate inhibitory profiles. Fraxin showed substantial inhibition of UGT1A1 (residual activity 31.9 ± 1.8%; inhibition 68.1%) and UGT1A6 (26.8 ± 1.9%; inhibition 73.2%), but minimal activity toward UGT1A9 (residual activity 90.0 ± 2.8%; inhibition 10.0%). Daphnetin showed moderate inhibition of UGT1A9 (residual activity 42.4 ± 1.7%; inhibition 57.6%), UGT1A6 (38.0 ± 0.7%; inhibition 62.0%), and UGT1A1 (52.6 ± 1.6%; inhibition 47.4%). Esculetin showed moderate inhibition of both UGT1A1 (residual activity 55.6 ± 1.1%; inhibition 44.4%) and UGT1A9 (58.3 ± 1.8%; inhibition 41.7%). Esculin exhibited the weakest overall inhibitory profile, with residual activities consistently above 65% across all 11 isoforms. None of these four compounds met the dual-isoform >80% inhibition criterion for progression to kinetic characterisation. The structural basis for these differential inhibitory profiles is discussed in Section 3.

**Figure 2:**
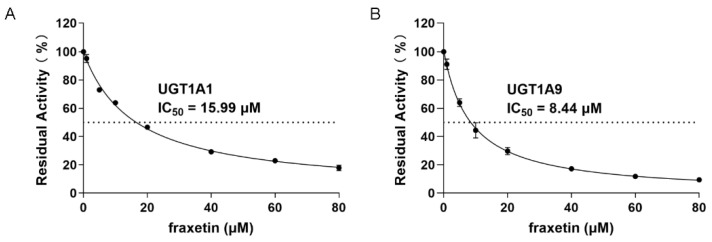
Concentration-dependent inhibition of fraxetin on UGT1A1 (**A**) and UGT1A9 (**B**). The dotted line indicates 50% residual activity. Data are represented as mean ± SE from triplicate experiments.

Concentration-dependent inhibition studies were conducted for fraxetin against UGT1A1 and UGT1A9 using 4-methylumbelliferone (4-MU) as the probe substrate (Figure 2). Fraxetin inhibited both isoforms in a concentration-dependent manner, with IC_50_ values of 15.99 μM (95% CI: 13.76–18.65 μM) for UGT1A1 and 8.44 μM (95% CI: 7.36–9.69 μM) for UGT1A9, indicating approximately 1.9-fold greater apparent potency toward UGT1A9 under the assay conditions employed. As IC_50_ values are inherently dependent on substrate concentration and inhibition mechanism, inhibition kinetic experiments were subsequently performed to determine the inhibition constants (*K*_i_) and characterise the inhibition type of fraxetin toward each isoform (Section 3).

### 2.2. Inhibition Kinetics

**Figure 3:**
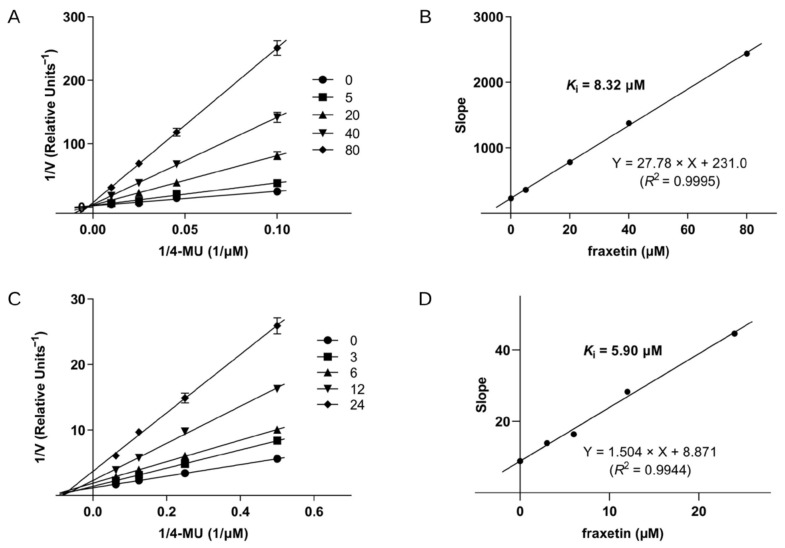
Lineweaver–Burk and Dixon plots for fraxetin inhibition of 4-MU glucuronidation in recombinant UGT1A1 (**A**,**B**) and UGT1A9 (**C**,**D**). The numbers in the legend represent the concentrations of fraxetin (μM). Points represent mean ± SE from triplicate measurements.

Full enzyme kinetic experiments were conducted to elucidate the inhibitory mechanisms of fraxetin toward UGT1A1 and UGT1A9 (Figure 3). The experimental velocity data at multiple substrate and inhibitor concentrations were fitted to four inhibition models (competitive, noncompetitive, uncompetitive, and mixed inhibition), and the best-fit model was selected based on goodness-of-fit criteria.

For UGT1A1, fitting to the four inhibition models identified the mixed inhibition model (Equation (4)) as the best fit, with a *K*_i_ value of 8.32 μM (*R*^2^ = 0.9995) and *α* = 3.77. In the Lineweaver–Burk plot, lines generated at different fraxetin concentrations converged in the second quadrant (negative 1/[*S*], positive 1/*v*), consistent with mixed-type inhibition in which fraxetin displays higher affinity for the free enzyme than for the enzyme–substrate complex (*α* > 1). The relatively large *α* value (3.77) indicates a preferentially competitive character, whereby binding of fraxetin to the free enzyme is substantially favoured over binding to the enzyme–substrate complex. Dixon plot analysis independently confirmed the *K*_i_ value, with the line intersections yielding an estimate in close agreement with the slope replot value (deviation < 2%), providing internal validation of the kinetic determination.

For UGT1A9, fitting to the four inhibition models similarly identified mixed inhibition (Equation (4)) as the best-fit model, with a *K*_i_ value of 5.90 μM (*R*^2^ = 0.9944) and *α* = 1.89. In the corresponding Lineweaver–Burk plot, lines at different fraxetin concentrations likewise converged in the second quadrant, confirming the preferentially competitive mixed-type character. Dixon plot analysis corroborated this result, and the IC_50_ predicted from the mixed inhibition model at [*S*] = *K*_m_ showed good agreement with the experimentally determined value (observed IC_50_ = 8.44 μM; deviation < 10%), confirming model-data consistency. Fraxetin inhibited UGT1A9 with approximately 1.4-fold higher potency than UGT1A1, as reflected by the respective *K*_i_ values (5.90 vs. 8.32 μM). All kinetic parameters are summarised in Table 1.

### 2.3. Molecular Docking and Protein–Ligand Interaction Profiling

To explore the structural basis of the isoform-selective inhibitory profiles observed in the screening, molecular docking, and PLIP interaction analysis were performed for fraxetin, fraxin, and daphnetin against UGT1A1 and UGT1A9. Because no experimental crystal structures of human UGT1A1 or UGT1A9 are currently available in the Protein Data Bank, AlphaFold Monomer v2.0 predicted structures for UGT1A1 (UniProt P22309) and UGT1A9 (UniProt O60656) were retrieved from the AlphaFold Protein Structure Database and used as receptor models for docking [18,19]. Fraxetin docked into the predicted substrate-binding pocket of UGT1A1 and UGT1A9 with binding free energies of −6.8 and −7.6 kcal/mol, respectively. Fraxin yielded more negative docking scores (−8.2 and −8.9 kcal/mol for UGT1A1 and UGT1A9, respectively), while daphnetin showed moderately reduced affinity (−6.3 and −7.2 kcal/mol). The binding free energies and in vitro inhibitory activities of all compounds are summarised in Table 2; detailed PLIP interaction data are provided in Table S2.

**Figure 4:**
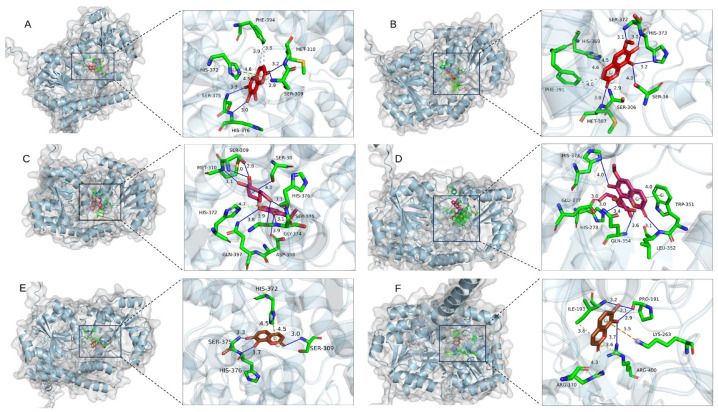
Molecular docking of fraxetin, fraxin, and daphnetin into the binding sites of UGT1A1 and UGT1A9. Fraxetin docked into UGT1A1 (**A**) and UGT1A9 (**B**); fraxin docked into UGT1A1 (**C**) and UGT1A9 (**D**); daphnetin docked into UGT1A1 (**E**) and UGT1A9 (**F**). The right panels show enlarged views of the binding pocket with key interacting residues. Protein–ligand interactions were identified and visualised using PLIP (Protein–Ligand Interaction Profiler), with interacting distances labelled in Ångströms (Å). Non-covalent interactions follow the PLIP (v2.4.0) colour scheme: hydrogen bonds (blue solid lines), hydrophobic interactions (grey dashed lines), salt bridges (yellow dashed lines), π-stacking (green dashed lines), and π-cation interactions (orange dashed lines).

PLIP analysis of the fraxetin complexes revealed structurally homologous but quantitatively distinct interaction networks. In UGT1A1, fraxetin formed four hydrogen bonds, two hydrophobic contacts with PHE394 (3.80 and 3.94 Å), one salt bridge, and one T-shaped π-stacking interaction with HIS372 (4.62 Å; 64.91°). The catechol O2 and O3 atoms engaged a contiguous Ser–Met–Ser–His hydrogen bond network (SER309/MET310→O2; SER375/HIS376→O3) spanning the catalytic binding site (Figure 4A). In UGT1A9, the conserved Ser–Met–Ser–His network was fully recapitulated, with the additional feature that HIS373 functioned as both hydrogen bond donor and acceptor toward O3—a bidirectional interaction absent in UGT1A1. PHE391 formed a single hydrophobic contact (3.95 Å), and HIS369 engaged a more compact T-shaped π-stacking interaction (4.52 Å; 74.17°) than its UGT1A1 counterpart (Figure 4B). The richer polar interaction network in UGT1A9 is consistent with its more favourable binding free energy and higher kinetic potency.

PLIP analysis of the fraxin complexes provided a structural explanation for the UGT1A9-specific inhibitory paradox. In UGT1A1 (Figure 4C), fraxin engaged several of the same protein-side residues as fraxetin (SER309, MET310, SER375, HIS376); however, these hydrogen bonds were directed toward glucose hydroxyl groups rather than the coumarin catechol O2/O3 atoms, indicating partial displacement of the coumarin scaffold from its catalytic position. Additional peripheral contacts with SER38, GLY374, ASP396, and GLN397, formed exclusively through glucose hydroxyls, collectively account for the more negative docking score. This partially productive binding geometry is consistent with the substantial but sub-maximal UGT1A1 inhibition observed in vitro (68.1%). In UGT1A9 (Figure 4D), the displacement was more complete: the glucose ring formed a parallel π-stacking interaction with TRP351 (3.98 Å; 8.76°), anchoring fraxin away from the catalytic network, with all hydrogen bonds migrating to peripheral residues (HIS278, LEU352, GLN354, GLU377) and complete loss of PHE391 hydrophobic contact and HIS369 salt bridge—fully consistent with the near-absence of UGT1A9 inhibitory activity (10.0%). For daphnetin, loss of the C-6 methoxy group eliminated PHE394/PHE391 hydrophobic contact in both isoforms. In UGT1A1 (Figure 4E), HIS372 salt bridge and π-stacking interactions were partially retained, consistent with the observed moderate inhibition (47.4%). In UGT1A9 (Figure 4F), daphnetin adopted a completely different orientation stabilised by ILE193, dual ARG salt bridges, and a π-cation interaction with LYS263—residues remote from the catalytic network—yet moderate inhibitory activity (57.6%) was still observed, suggesting that peripheral binding can partially restrict substrate access. These observations indicate that productive catalytic engagement is governed by the positioning of the coumarin nucleus rather than the total interaction count, and the structural determinants are discussed mechanistically in Section 3.

### 2.4. Molecular Dynamics Simulations and MM/GBSA/PBSA Binding Free Energy

**Figure 5:**
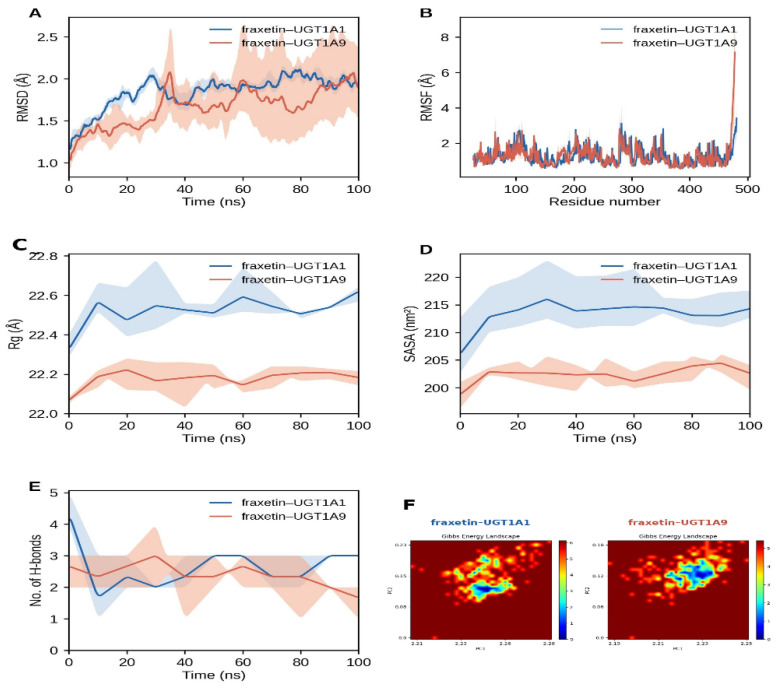
Molecular dynamics (MD) simulation analysis of UGT1A1–fraxetin (blue) and UGT1A9–fraxetin (red) complexes over 100 ns; solid lines show the mean and shaded regions the SD across three independent replicates. (**A**) Root mean square deviation (RMSD) of the protein backbone; (**B**) root mean square fluctuation (RMSF) of residues; (**C**) radius of gyration (Rg); (**D**) solvent-accessible surface area (SASA); (**E**) number of hydrogen bonds between fraxetin and protein; (**F**) Gibbs free energy landscape on the PC1–PC2 coordinates for UGT1A1–fraxetin (**left**) and UGT1A9–fraxetin (**right**).

Three independent 100 ns molecular dynamics (MD) replicates were performed for each of the fraxetin–UGT1A1 and fraxetin–UGT1A9 complexes to evaluate binding stability and dynamic behaviour (Figure 5). Backbone RMSD analysis (Figure 5A) showed that both complexes reached equilibration within the first ~30 ns and maintained overlapping backbone RMSD values in the range of approximately 1.8–2.0 Å over the remainder of the 100 ns trajectory, indicating comparable global structural stability of the two systems. Although the UGT1A9 trajectory exhibited somewhat larger transient excursions between 30–70 ns, these returned to baseline and did not represent persistent structural drift, indicating stable global protein dynamics. Ligand-centred analysis showed that fraxetin remained continuously bound and deeply buried within the protein in both complexes (on average 88% and 93% of ligand heavy atoms maintaining a protein heavy-atom contact within 4 Å for UGT1A1 and UGT1A9, respectively, with the closest ligand–protein distance remaining approximately 2.9 Å throughout). However, the ligand heavy-atom RMSD relative to the docking-derived pose reached 4–11 Å (per-isoform means 7.4 Å and 8.9 Å), indicating that fraxetin equilibrated to bound conformation(s) distinct from the initial docked pose rather than retaining that pose. Residue-level flexibility, radius of gyration, solvent-accessible surface area and hydrogen bonding remained stable and comparable between the two isoforms across the trajectory (Figure 5B–F).

**Figure 6:**
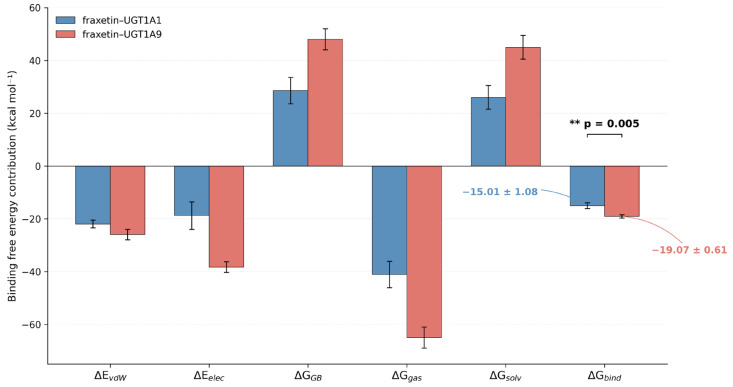
MM/GBSA binding free energy components for the fraxetin–UGT1A1 (blue) and fraxetin–UGT1A9 (red) complexes, averaged across three independent 100 ns MD replicates. Bars show mean ± SD for the van der Waals (ΔE_vdW_), electrostatic (ΔE_elec_), Generalised-Born polar solvation (ΔG_GB_), gas-phase (ΔG_gas_), total solvation (ΔG_solv_) and total binding (ΔG_bind_) free energy contributions. Welch’s two-sided *t*-test on ΔG_bind_: ** *p* = 0.005.

Binding free energies were calculated by both the Generalised Born (MM/GBSA) and Poisson–Boltzmann (MM/PBSA) implicit-solvent models on the equilibrated trajectory segment of each of three independent replicates (Figure 6). MM/GBSA, which is generally considered more reliable than MM/PBSA for binding interfaces dominated by strong electrostatic contacts, yielded mean binding free energies that were significantly more negative for fraxetin–UGT1A9 (−19.07 ± 0.61 kcal/mol; CV = 3.2%) than for fraxetin–UGT1A1 (−15.01 ± 1.08 kcal/mol; CV = 7.2%), with an inter-isoform difference in ΔΔ*G* = −4.06 kcal/mol that was statistically significant by Welch’s *t*-test (*t* = 5.69, *p* = 0.005, *n* = 3). The corresponding MM/PBSA estimates were less discriminating between the two isoforms (UGT1A1: −13.59 ± 1.38 kcal/mol; UGT1A9: −13.66 ± 2.14 kcal/mol; ΔΔ*G* ≈ 0.07 kcal/mol, *p* = 0.96), reflecting the well-documented tendency of the Poisson–Boltzmann solver to overestimate the polar desolvation penalty for binding interfaces with strong electrostatic interactions. In the present systems, fraxetin–UGT1A9 displayed a substantially stronger gas-phase electrostatic interaction than fraxetin–UGT1A1 (mean Δ*E*_EL_ = −38.3 vs. −18.8 kcal/mol), of which approximately 24 kcal/mol was offset by an enlarged polar-solvation term Δ*E*_PB_ in the MM/PBSA calculation, masking the underlying selectivity. MM/GBSA was therefore adopted as the primary energetic descriptor for this study, and MM/PBSA values are reported alongside for methodological transparency. The MM/GBSA-derived selectivity (ΔΔ*G* = −4.06 kcal/mol) is directionally concordant with the docking-derived selectivity (ΔΔ*G* = −0.80 kcal/mol) and the kinetic estimate from the *K*_i_ ratio (ΔΔ*G* ≈ −0.20 kcal/mol). Although the absolute magnitudes differ across methods—as is expected given their distinct theoretical foundations and the known tendency of MM/GBSA to over-amplify electrostatically driven selectivity—all three approaches identify UGT1A9 as the preferred binder, providing convergent cross-method support for the experimentally observed inhibition pattern.

Per-residue MM/GBSA energy decomposition was attempted but yielded large inter-replicate variability for individual residues (typical SD ≥ |mean| for the principal contributors), reflecting the well-recognised difficulty of converging single-residue contributions on the 100 ns timescale; quantitative per-residue values are therefore not reported, and the residue-level interpretation of the binding interface is provided by the docking-based PLIP analysis presented in Section 2.3.

### 2.5. In Vitro–In Vivo Extrapolation and Herb–Drug Interaction Risk Prediction

The potential for clinically significant herb–drug interactions (HDI) mediated by fraxetin inhibition of UGT1A1 and UGT1A9 was assessed using the FDA 2020 static model for in vitro–in vivo extrapolation (IVIVE). The systemic inhibition risk ratio (*R*_1_) was calculated as *R*_1_ = 1 + [*I*]_max,u_/*K*_i_, where [*I*]_max,u_ = *f*_u,p_ × *C*_max_ represents the maximum unbound systemic plasma concentration, and *K*_i_ is the inhibition constant determined in vitro. In the absence of clinical pharmacokinetic data for fraxetin in humans, published animal *C*_max_ values were used as pharmacokinetic surrogates: 10.80 μM in rats following oral administration of fraxin at 50 mg/kg, 2.40 μM in rats following fraxetin at 25 mg/kg, and 1.78 μM in beagle dogs. A worst-case assumption of *f*_u,p_ = 1.0 (i.e., no plasma protein binding) was applied in accordance with FDA guidance, which recommends this approach when human plasma protein binding data are unavailable. Because *f*_u,inc_ correction for fraxetin was not determined experimentally, *K*_i_ values were used directly (*K*_i,u_ ≈ *K*_i_), which represents a conservative approach consistent with the low lipophilicity of fraxetin and the low protein concentrations used in the incubation system.

*R*_1_ exceeded the FDA threshold of 1.02 under all animal *C*_max_ scenarios evaluated (Table 3). At the highest *C*_max_ scenario (rat, fraxin 50 mg/kg; *C*_max_ = 10.80 μM), *R*_1_ was 2.30 for UGT1A1 and 2.83 for UGT1A9. At the lowest *C*_max_ scenario (beagle dog; 1.78 μM), *R*_1_ values of 1.21 (UGT1A1) and 1.30 (UGT1A9) still exceeded the threshold. The higher *R*_1_ values for UGT1A9 across all scenarios are consistent with the lower *K*_i_ of fraxetin toward UGT1A9 (5.90 vs. 8.32 μM for UGT1A1), confirming that UGT1A9 represents the higher-priority isoform for HDI concern at systemic exposure levels.

The intestinal inhibition risk ratio (*R*_1,gut_) was calculated as *R*_1,gut_ = 1 + *I*_gut_/*K*_i_, where *I*_gut_ = Dose/250 mL represents the estimated intestinal luminal concentration following oral administration. The relevant intestinal fraxetin dose was derived from the Chinese Pharmacopoeia daily Cortex Fraxini dose of 6–12 g: fraxin (the abundant 8-O-glucoside, 2–5% dry weight, 120–600 mg) is hydrolysed essentially completely to fraxetin by gut β-glucosidases (molecular-weight conversion 208/370 ≈ 0.56, 67–337 mg), with a further 6–60 mg contributed by the aglycone present directly in the bark (0.1–0.5%), giving a total effective daily intestinal fraxetin exposure of approximately 75 mg (low), 150 mg (typical) and 375 mg (high). Because fraxetin is sparingly soluble in aqueous media (approximately 0.09 mg mL^−1^, ≈0.43 mM) [20], its luminal free concentration is solubility-limited rather than dose-proportional: the nominal *I*_gut_ = Dose/250 mL for these doses (≈1.4–7.2 mM) exceeds the saturation concentration, so the physiologically attainable Igut is capped at ≈0.43 mM [9]. Applying this solubility-capped value, *R*_1,gut_ = 1 + *I*_gut_/*K*_i_ ≈ 53 for UGT1A1 and ≈74 for UGT1A9—still approximately five- to seven-fold above the FDA high-risk threshold of 11. This intestinal signal is robust to conservative correction: it would fall below the threshold only if the attainable luminal concentration dropped below ≈60 μM, well under the measured solubility, and the simultaneous application of the solubility cap, a realistic plasma-protein-binding correction and the observed mixed-type (rather than competitive) mechanism still leaves *R*_1,gut_ above 11. We therefore adopt the solubility-capped *R*_1,gut_ (≈53–74) as the physiologically relevant intestinal estimate, in preference to the dose-proportional values (of order 10^3^) that assume complete dissolution. These results indicate that co-administration of Cortex Fraxini-containing preparations with drugs primarily metabolised by hepatic or intestinal UGT1A1 or UGT1A9 may carry a clinically meaningful risk of pharmacokinetic HDI. UGT1A9 substrates of particular clinical relevance include propofol, canagliflozin, dapagliflozin, and ertugliflozin, among others; UGT1A1 substrates include irinotecan (via its active metabolite SN-38) and bilirubin. Several important limitations should be acknowledged when interpreting these predictions. First, the *R*_1_ and *R*_1,gut_ values reported here are derived from animal pharmacokinetic data in the absence of human *C*_max_ values for fraxetin; interspecies differences in absorption, distribution, and first-pass metabolism may render these estimates non-conservative or overly conservative depending on the direction of translation. Second, the inhibition constants were determined using recombinant UGT isoforms in a defined buffer system, which may not fully recapitulate the complexity of intestinal and hepatic microsomal environments in vivo. Third, the mixed-type inhibition mechanism (*α* > 1) identified for both isoforms means that the inhibitory effect of fraxetin is attenuated in the presence of the substrate; the *R*_1_ model assumes competitive inhibition for simplicity and may therefore overestimate risk. Notwithstanding these limitations, the magnitude of *R*_1,gut_ values—exceeding the FDA threshold by approximately five- to seven-fold—indicates that intestinal HDI risk warrants clinical investigation, particularly for Cortex Fraxini preparations co-administered with narrow therapeutic index UGT1A1 or UGT1A9 substrates.

## 3. Discussion

To our knowledge, this is the first study to systematically map the UGT-inhibition profile of the principal Cortex Fraxini coumarins across the human UGT panel and to translate that profile into a solubility-constrained, compartment-resolved interaction risk, identifying the intestinal lumen rather than the systemic circulation as the site at which inhibition is predicted to be clinically relevant. To clarify the incremental contribution of this work, Table 4 contrasts what was previously established regarding the UGT-inhibitory potential of Cortex Fraxini coumarins with the findings of the present integrated study. Earlier work characterised fraxetin chiefly as a UGT1A9 substrate and as the predominant circulating coumarin after oral administration, but its inhibitory action toward human UGT isoforms had not been evaluated systematically, no inhibition constants or mechanism had been reported, no structural binding model existed, and no exposure-based clinical-risk assessment had been performed. The conceptual advance is therefore not the application of any single technique but the integration of profiling, mechanism and exposure into one compartment-resolved risk statement: the data relocate the predicted interaction from the systemic circulation, where it is borderline, to the intestinal lumen, where it is robust.

Among the five compounds examined, fraxetin was the only one to inhibit both UGT1A1 and UGT1A9 by more than 80%, qualifying it as the primary UGT-active component of Cortex Fraxini under the screening conditions, whereas the analogues showed isoform-selective or only moderate profiles. Fraxetin represents the first UGT inhibitor reported within the 7,8-dihydroxy-6-methoxycoumarin chemotype, a structural class distinct from the flavonoid (icaritin, scutellarin), flavanonol (silybin) and flavone (quercetin) inhibitors that dominate the existing literature on natural-product UGT1A9 inhibition. Beyond compound novelty, three translational insights distinguish this work: (i) the C-6 methoxy/7,8-dihydroxycoumarin dual-pharmacophore model, derived here from the comparative analysis of five structurally related Cortex Fraxini constituents, provides a structure–activity framework for the wider coumarin chemical space that has not previously been formulated for UGT1A9; (ii) the convergence of kinetic (*K*_i_ ratio = 1.41), docking (ΔΔ*G* = −0.80 kcal/mol) and triplicate MM/GBSA (ΔΔ*G* = −4.06 kcal/mol, *p* = 0.005) provides cross-method, directionally concordant support for the UGT1A9 preference, an integrated kinetic–structural assessment seldom reported in the natural-product UGT literature; and (iii) the simultaneously elevated systemic (*R*_1_ = 1.21–2.83) and intestinal (*R*_1,gut_ solubility-capped ≈ 53–74) screening-tier risk signal under FDA static-model assessment positions fraxetin as a priority candidate for clinical herb-drug interaction studies with UGT1A1- and UGT1A9-substrate drugs of narrow therapeutic index, contextualising the in vitro findings within a regulatory-grade DDI prediction framework.

Full inhibition kinetic characterisation identified mixed-type (preferentially competitive) inhibition for fraxetin against both UGT1A1 (*K*_i_ = 8.32 μM, *α* = 3.77) and UGT1A9 (*K*_i_ = 5.90 μM, *α* = 1.89). Mixed-type inhibition, in which an inhibitor retains finite affinity for both the free enzyme and the enzyme–substrate complex, has been reported for numerous natural product UGT inhibitors and is consistent with the broad accommodating architecture of the UGT substrate-binding pocket [21]. The observed IC_50_-based selectivity ratio (IC_50,1A1_/IC_50,1A9_ = 1.89) is higher than the *K*_i_-based ratio (*K*_i,1A1_/*K*_i,1A9_ = 1.41), a discrepancy that is mathematically expected rather than mechanistically anomalous. For a mixed-type inhibitor at [*S*] = *K*_m_, the Cheng–Prusoff relationship extends to IC_50_ = *K*_i_ × 2*α*/(*α* + 1) [22,23], so the IC_50_/*K*_i_ expansion factor is *α*-dependent (2 × 3.77/4.77 = 1.58 for UGT1A1; 2 × 1.89/2.89 = 1.31 for UGT1A9). Because the larger *α* value of UGT1A1 produces a proportionally greater IC_50_ expansion relative to *K*_i_, the IC_50_-derived selectivity ratio exceeds the *K*_i_-derived ratio. This relationship provides internal validation that the kinetic parameters are mutually consistent. The lower *α* value for UGT1A9 (1.89 vs. 3.77 for UGT1A1) indicates that fraxetin’s affinity advantage for the free enzyme over the enzyme–substrate complex is less pronounced in UGT1A9. While the structural basis for this difference cannot be definitively established from the present data, it may be related to the richer polar interaction network identified by PLIP analysis in the UGT1A9 complex, which could facilitate partial retention of ligand contacts when substrate is present. This interpretation is speculative and requires further investigation. Benchmarked against established UGT inhibitors profiled with the same 4-methylumbelliferone probe (Table S3), fraxetin falls within the moderate-potency band. Its inhibition constants (*K*_i_ = 8.3 μM for UGT1A1 and 5.9 μM for UGT1A9) are one to three orders of magnitude weaker than those of the prototypical potent inhibitors of each isoform—niflumic acid for UGT1A9 (*K*_i_ ≈ 0.03 μM) and atazanavir or kinase inhibitors for UGT1A1 (IC_50_ 0.13–0.42 μM)—yet are comparable to those of dietary and herbal UGT inhibitors for which intestinal herb–drug interaction signals have been reported, including norathyriol, ginsenoside Rc and the milk-thistle flavonolignans. Characterising fraxetin as a moderate-potency inhibitor therefore reflects its absolute potency accurately, whereas its clinical relevance derives not from per-enzyme potency but from the high luminal concentrations attainable in the intestinal compartment, as quantified by the IVIVE analysis.

Docking and PLIP analysis rationalised the screening profile through a dual-element pharmacophore. The highest potency required both the 7,8-dihydroxycoumarin aglycone engaging the conserved Ser–Met–Ser–His hydrogen-bond network and the C-6 methoxy group providing PHE-mediated hydrophobic anchoring; fraxetin satisfies both and was the most potent compound. The two isoforms differ in stringency: in UGT1A9, loss of either element (C-8 glycosylation in fraxin, or absence of the C-6 methoxy in daphnetin) abolished marked inhibition, whereas the more accommodating UGT1A1 pocket retained partial activity (fraxin ~68%, daphnetin ~47%, esculetin ~44%) through peripheral contacts. The UGT1A9 fraxin paradox—high docking affinity (Δ*G* = −8.9 kcal/mol) but near-absent inhibition (~10%)—was resolved by a UGT1A9-specific TRP351 π-stacking interaction with the glucose ring that anchors fraxin away from the catalytic network, an interaction absent in UGT1A1. Molecular dynamics with MM/GBSA across three replicates gave directionally concordant support for the UGT1A9 preference (ΔΔ*G* = −4.06 kcal/mol, *p* = 0.005), whereas MM/PBSA did not discriminate the isoforms (ΔΔ*G* ≈ 0.07 kcal/mol, *p* = 0.96) owing to its larger polar-solvation penalty at the strongly electrostatic UGT1A9 interface; MM/GBSA was therefore adopted as the primary descriptor, consistent with the active-site flexibility documented for UGT isoforms [24,25] and the transient hydrogen-bond exchange typical of protein–ligand simulations [26]. Because the difference arose from an ensemble of bound conformations rather than a single retained pose, it is treated as directional cross-method corroboration of the kinetic and docking rankings—the three methods agree in direction though not magnitude, as expected for polar binding sites [27]—rather than as the keystone of the selectivity argument. Residue-level interpretation, therefore, rests on the docking/PLIP analysis (Section 2.3); per-residue MM/GBSA decomposition was insufficiently converged on the 100 ns timescale and is not reported quantitatively [27]. IVIVE analysis using the FDA 2020 static model indicates that fraxetin carries a meaningful potential for HDI via inhibition of both UGT1A1 and UGT1A9. Systemic *R*_1_ values exceeded the FDA threshold of 1.02 under all pharmacokinetic scenarios tested (range: 1.21–2.83), and intestinal *R*_1,gut_ values of approximately 53 (UGT1A1) and 74 (UGT1A9), after capping the luminal concentration at fraxetin aqueous solubility, exceeded the high-risk threshold of 11 by approximately five- to seven-fold (*R*_1,gut_/threshold = 53/11 and 74/11, respectively). The scale of the intestinal signal arises from the high luminal concentration (*I*_gut_ = capped at the aqueous solubility, ≈0.43 mM) relative to the *K*_i_ values (5.90–8.32 μM), and would remain pharmacologically significant even at oral doses an order of magnitude lower. UGT1A1 substrates of particular clinical concern include SN-38, the active metabolite of irinotecan, whose glucuronidation by UGT1A1 is the principal detoxification mechanism and whose impairment is associated with severe diarrhoea and neutropenia [28,29]. UGT1A9 substrates of clinical relevance include propofol and the SGLT2 inhibitors dapagliflozin and canagliflozin, which undergo primary or substantial glucuronidation via UGT1A9 [30,31]. Based on pharmacokinetic data in the literature, intestinal UGT1A9 is expected to contribute to first-pass glucuronidation of these agents, and inhibition of intestinal UGT1A9 may therefore theoretically alter systemic exposure—though this extrapolation extends beyond the scope of the present in vitro data and requires clinical investigation to confirm [1]. These findings are consistent with HDI risk assessments reported for other natural product UGT inhibitors, including silybins from milk thistle, for which in vitro IVIVE predictions of UGT1A1-mediated SN-38 interactions have been demonstrated [14].

Several clinical UGT1A1 and UGT1A9 substrate drugs of particular concern in the context of co-administration with fraxetin-containing Cortex Fraxini preparations are summarised in Supplementary Table S4. For UGT1A1, the predicted increase in AUC for the irinotecan active metabolite SN-38 ranges from 21% to 130% at the systemic *R*_1_ of 1.21–2.30 obtained for UGT1A1 in this study; this would be clinically significant given the dose-limiting toxicity of SN-38 (severe diarrhoea, neutropenia, particularly in UGT1A1*28 polymorphism carriers) [28,29]. The HIV integrase inhibitor raltegravir, dependent on UGT1A1 for approximately 70% of clearance [32], would similarly be expected to show clinically meaningful exposure increases. For UGT1A9, propofol (whose hepatic glucuronidation is a major clearance pathway), the immunosuppressant mycophenolic acid (glucuronidated primarily by UGT1A9 and UGT2B7), and the multikinase inhibitor sorafenib all have narrow therapeutic indices and predictable consequences from impaired UGT1A9 clearance [33]. The clinical relevance of these predicted interactions warrants pharmacokinetic verification before Cortex Fraxini-containing preparations are co-administered with such agents.

Although no epidemiological prevalence data exist for direct Cortex Fraxini–conventional drug co-administration, an indication-overlap analysis identifies a structural mechanism for co-exposure. The principal clinical indications of Cortex Fraxini in the Chinese Pharmacopoeia (2020) [5]—damp-heat dysentery, gout and hyperuricaemia, and ophthalmic inflammation—correspond to patient populations who concurrently receive UGT1A1 and UGT1A9 substrate drugs. Gout and hyperuricaemia patients in particular frequently receive febuxostat (a UGT1A1/1A3/1A9 substrate) and NSAIDs such as indomethacin and diclofenac that are extensively glucuronidated by UGT1A9 and/or UGT1A1. Cortex Fraxini is itself a component of anti-gout polyherbal formulations (e.g., the Qinpi Tongfeng formula, evaluated in randomised controlled trials and recommended in clinical guidelines for acute gouty arthritis [34]), placing it in a clinical setting in which parallel use of conventional anti-gout pharmacotherapy is common. The indication-driven co-exposure risk is therefore substantial and identifiable from existing prescribing patterns, even in the absence of direct prevalence statistics.

Two further considerations support the intestinal-exposure argument. First, the high luminal concentrations are not transient: following first-order absorption, the time-averaged intestinal fraxetin concentration over the 0–2 h post-dose window remains in the high-micromolar-to-low-millimolar range (approximately 0.6 × initial *I*_gut_), and, because the Pharmacopoeia daily dose is administered in two to three divided doses, an inhibitory intestinal concentration is maintained for much of the dosing day. Second, although fraxetin is itself a UGT1A9 substrate and might in principle be depleted by the enzymes it inhibits, meaningful self-depletion is unlikely: the molar amount delivered in a single dose (≈0.4–2.4 mmol for 75–500 mg) vastly exceeds the saturable glucuronidation capacity of intestinal UGT1A9 in the immediate post-dose window, and the concentration required for half-maximal inhibition (IC_50_ 8.44 μM) is approximately fifty-fold below the solubility-capped *I*_gut_, so the intestinal inhibitor pool is not materially eroded over the relevant timescale. The intestinal *R*_1,gut_ signal is therefore robust to both temporal dilution and substrate self-clearance.

A further spatial consideration refines, but does not overturn, the intestinal risk estimate. Because fraxin is absorbed poorly in its intact glucoside form and is hydrolysed to fraxetin chiefly by mucosal and microbial β-glucosidases, liberation of the active aglycone is not uniform along the gastrointestinal tract: it is limited in the proximal jejunum and increases distally toward the ileum and colon, where microbial β-glucosidase activity is highest. Intestinal UGT1A1 and UGT1A9 expression, by contrast, is generally greater in the proximal small intestine. The intestinal *R*_1,gut_ therefore represents a segment-averaged worst-case in which the regions of maximal inhibitor liberation and maximal victim-drug glucuronidation only partially overlap, and the true magnitude of any intestinal interaction will depend on the regional co-localisation of fraxetin release and substrate-drug absorption. Capturing this heterogeneity would require a spatially resolved, segment-based intestinal PBPK model, which we identify as a priority for future refinement.

Clinical herb-drug interaction risk arising from fraxetin-mediated UGT inhibition is expected to be amplified in several patient subgroups. Patients carrying the UGT1A1*28 polymorphism (homozygous *28/*28; Gilbert’s syndrome phenotype, ~10% Caucasians and 3–5% East Asians [35]) have baseline UGT1A1 activity reduced to approximately 30% of wild-type [29] and are already at increased risk of SN-38 toxicity during irinotecan therapy; concomitant UGT1A1 inhibition by fraxetin in such patients would be expected to compound this risk. Similarly, UGT1A9*22 (T-275A) and UGT1A9*3 polymorphisms have been associated with altered mycophenolic acid pharmacokinetics [36]. Elderly patients exhibit approximately 25% reduction in hepatic UGT activity due to age-related decreases in microsomal protein content [36], and patients with moderate-to-severe hepatic impairment (Child–Pugh B–C) show further reductions in UGT-mediated clearance [36]. Sex-specific differences have also been reported, with female patients showing on average ~20% lower UGT1A9 expression than males [36]. Cortex Fraxini is commonly co-administered with multiple traditional herbal formulae and conventional medications in clinical practice, increasing the likelihood of clinically observable herb-drug interactions in real-world polypharmacy contexts. Particular caution is therefore warranted when fraxetin-containing preparations are administered to patients receiving narrow-therapeutic-index UGT substrates, especially those with the genetic, age-, sex-, or hepatic-impairment-related vulnerabilities outlined above (Supplementary Table S4).

**Critical evaluation of limitations and their quantitative impact.** The principal limitations are stated below with their likely direction and magnitude of effect. (i) Inhibition constants were measured with recombinant enzymes rather than hepatic or intestinal microsomes and without albumin supplementation, which can shift the apparent UGT1A9 *K*_i_ by up to roughly two-fold in either direction. (ii) The plasma unbound fraction was conservatively set to *f*_u,p_ = 1.0; correction to a realistic value can only lower the systemic risk ratio, by at most the 1/*f*_u,p_ factor (plausibly ~2–3-fold for a moderately protein-bound coumarin), so the borderline systemic signal (*R*_1_ ≈ 1.2–2.8) may fall below unity after correction, whereas the intestinal signal is unaffected. (iii) The FDA *R*_1_ model assumes competitive inhibition, whereas fraxetin displayed mixed-type kinetics (*α* > 1), so systemic risk is, if anything, over-estimated; because intestinal *R*_1,gut_ exceeds the threshold of 11 by approximately five- to seven-fold even after solubility capping, the qualitative intestinal conclusion is unlikely to be reversed by plausible refinements. (iv) Structural inferences rest on AlphaFold apo models and an MD ensemble in which the ligand deviated from the docked pose (RMSD 4–11 Å) and the secondary MM/PBSA descriptor did not statistically resolve isoform selectivity (ΔΔ*G*, *p* = 0.96), and the preference indicated by the primary MM/GBSA descriptor (*p* = 0.005) should be read as directional rather than quantitative; residue-level contacts are therefore hypotheses for co-crystallographic or mutagenesis testing rather than established determinants. (v) The intestinal inhibitor concentration I_gut_ is an upper-bound luminal estimate that does not model the spatial heterogeneity of fraxin-to-fraxetin hydrolysis, intestinal transit or dilution; together with the known tendency of the FDA static model to over-predict gut UGT interactions, this means the intestinal signal should be regarded as a first-tier hazard flag requiring dynamic PBPK and clinical confirmation. (vi) Fraxetin is itself a substrate of UGT1A9 [11]; depletion of the inhibitor through glucuronidation at long incubation times can underestimate potency, so the UGT1A9 *K*_i_ (5.90 μM) is a conservative upper bound. (vii) IVIVE relied on animal pharmacokinetic surrogates in the absence of human *C*_max_ data, adding interspecies extrapolation uncertainty to the systemic estimate, and the FDA static model is known to over-predict intestinal UGT interactions for rapidly cleared reversible inhibitors (a static model predicted a 5-fold raloxifene AUC increase for silibinin that was not observed clinically [30]). Notwithstanding these limitations, the convergence of inhibition kinetics, structural analysis, molecular dynamics and IVIVE across independent methodologies supports a coherent picture of fraxetin as the principal UGT-inhibitory component of Cortex Fraxini and prioritises UGT1A1 and UGT1A9 for clinical HDI assessment; the contribution of sulfotransferase-mediated metabolism to fraxetin elimination, and its potential to compensate for UGT inhibition, remain to be determined.

## 4. Materials and Methods

### 4.1. Chemicals and Reagents

**Figure 7:**
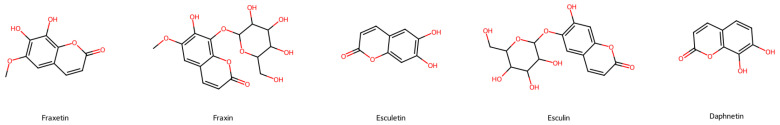
Chemical structures of the five major coumarin constituents of Cortex Fraxini examined in this study: the aglycones fraxetin, esculetin and daphnetin, and the glucosides fraxin (fraxetin 8-O-glucoside) and esculin (esculetin 6-O-glucoside). Structures are drawn in standard skeletal representation (carbon, black; oxygen, red; hydrogens implicit).

Fraxetin, fraxin, esculetin, esculin, and daphnetin (all ≥98% purity) were purchased from Solarbio Science & Technology Co., Ltd. (Beijing, China). Daphnetin (7,8-dihydroxycoumarin), a structural analogue of fraxetin lacking the C6-methoxy group, was included as a structural comparator for molecular docking analysis to examine the structural factors influencing UGT inhibition. Recombinant human UGT isoforms (UGT1A1, UGT1A3, UGT1A6, UGT1A7, UGT1A8, UGT1A9, UGT1A10, UGT2B4, UGT2B7, UGT2B15, and UGT2B17) were obtained from BD Gentest (Woburn, MA, USA). Uridine 5′-diphosphoglucuronic acid trisodium salt (UDPGA), 4-methylumbelliferone (4-MU), 4-methylumbelliferone-β-D-glucuronide (4-MUG), and all other chemicals were purchased from Sigma-Aldrich (St. Louis, MO, USA) unless otherwise noted. All solvents and reagents were of HPLC or analytical grade. Stock solutions of all test compounds were prepared in dimethyl sulfoxide (DMSO) and stored at −20 °C. The final DMSO concentration in all incubations was ≤0.1% (*v*/*v*). The chemical structures and approximate Cortex Fraxini content of the five constituents are summarised in Figure 7 and Table 5 [5]. The five compounds examined were selected to represent the principal coumarin constituents of Cortex Fraxini together with their biotransformation relationship. Esculin and fraxin are the two most abundant constituents [37] and occur predominantly as the 6-O- and 8-O-β-D-glucosides, respectively; both are hydrolysed by intestinal β-glucosidases to the aglycones esculetin and fraxetin. Fraxetin, although only a minor direct constituent of the bark, is the predominant coumarin recovered in the systemic circulation after oral dosing [38] and was therefore prioritised as the most exposure-relevant species. Daphnetin, a positional isomer of esculetin, was included to probe the influence of the catechol substitution pattern. This panel thus captures both the high-abundance glucoside prodrugs that govern intestinal exposure and the circulating aglycone that governs systemic exposure.

### 4.2. UGT Inhibition Screening

The 11 UGT isoforms (UGT1A1, 1A3, 1A6, 1A7, 1A8, 1A9, 1A10, 2B4, 2B7, 2B15, and 2B17) selected represent the major hepatic and intestinal drug-metabolising UGTs collectively responsible for the glucuronidation of the majority of clinical drugs undergoing Phase II metabolism [1,4], consistent with established inhibition-screening panels used in the regulatory and academic UGT literature. Pseudogenes (UGT1A2P, UGT1A12P), isoforms with markedly restricted tissue expression or limited characterised drug substrates (UGT1A5, UGT3A1, UGT3A2, UGT8A1), and isoforms for which validated recombinant enzyme is not commercially available were not included in the present panel [4].

The inhibitory effects of four main coumarin components of Cortex Fraxini—fraxetin, fraxin, esculetin, and esculin—and one structural analogue, daphnetin, on recombinant human UGT isoforms (UGT1A1, UGT1A3, UGT1A6, UGT1A7, UGT1A8, UGT1A9, UGT1A10, UGT2B4, UGT2B7, UGT2B15, and UGT2B17) were evaluated using 4-MU as a universal probe substrate, following previously described methods [39]. A typical 200 μL incubation mixture included recombinant UGT protein, 5 mM UDPGA, 10 mM MgCl_2_, and 50 mM Tris-HCl buffer (pH 7.4), with each test compound at a final concentration of 100 μM or a vehicle control (0.1% DMSO). For each isoform, the 4-MU concentration matched its reported apparent *K*_m_ value [40]. Incubation conditions followed those described previously by Li et al. [39]. After a 5 min pre-incubation at 37 °C, the reaction was initiated by adding UDPGA. Reactions were terminated by adding 200 μL of ice-cold acetonitrile containing 7-hydroxycoumarin (100 μM) as an internal standard. The mixtures were centrifuged at 12,000× *g* for 10 min at 4 °C. The supernatants were analysed by HPLC (Waters Corp., Milford, MA, USA) on a C18 column (4.6 mm × 250 mm, 5 μm, GL Sciences, Tokyo, Japan) at a flow rate of 1 mL/min, with UV detection at 316 nm. The mobile phase consisted of acetonitrile (A) and 0.5% formic acid aqueous solution (B), with a gradient elution: 0–5.5 min, 10–65% A; 5.5–9 min, 65% A; 9–10.5 min, 65–10% A; 10.5–12.5 min, 10% A. Residual UGT activity (%) was calculated relative to the vehicle control. All assays were performed in triplicate. For each isoform, the residual activities of the five test compounds and the vehicle control (n = 3 per group) were compared by two-way ANOVA (isoform × compound; ordinary, *α* = 0.05) followed by Dunnett’s multiple-comparison test against the vehicle control within each isoform (one family per row; multiplicity-adjusted *p* values). As fraxetin is itself a UGT substrate, partial glucuronidation during incubation may result in underestimation of inhibitory potency; this conservative bias is consistent with the risk-protective intent of IVIVE-based herb–drug interaction assessment. Goodness-of-fit was assessed by *R*^2^ > 0.95 for all fits; inter-assay coefficient of variation (CV) for IC_50_ estimates was <15% across three independent experiments. The screening concentration of 100 μM was selected per FDA drug–drug interaction guidance for initial inhibitor screening panels, which recommends this concentration to maximise inhibitor detection sensitivity. Physiological relevance of this concentration is further contextualised in the IVIVE section (Section 4.7) using estimated in vivo exposure parameters and sensitivity analyses across *C*_max_ scenarios.

### 4.3. Concentration-Dependent Inhibition and IC_50_ Determination

For UGT isoforms with 80% or greater inhibition in the primary screening, concentration-dependent inhibition studies were then conducted. The final concentrations of fraxetin tested were 0, 1, 5, 10, 20, 40, 60, and 80 μM against both UGT1A1 and UGT1A9. Incubation conditions, sample processing, and HPLC analysis were the same as those described in Section 4.2. IC_50_ values were determined by nonlinear regression analysis using GraphPad Prism 8.0 (GraphPad Software, La Jolla, CA, USA) with 95% confidence intervals. All assays were performed in triplicate.

### 4.4. Inhibition Kinetic Analysis

For UGT isoforms showing 80% or more inhibition in the primary screening, additional inhibition kinetic parameters were determined using a full substrate-inhibitor matrix design. The substrate concentration ranged from 0.5 × *K*_m_ to 4 × *K*_m_, while the inhibitor concentration ranged from IC_50_/4 to 4 × IC_50_, based on previously obtained *K*_m_ and IC_50_ values. Incubation conditions and sample processing were performed according to the procedures described in Section 4.2. The inhibition constant (*K*_i_) was calculated using nonlinear regression analysis based on four inhibition models: competitive (Equation (1)), noncompetitive (Equation (2)), uncompetitive (Equation (3)), and mixed inhibition (Equation (4)) [23]

(1) $$V=\frac{V_{max}[S]}{K_{m}(1+\frac{\left[I\right]}{K_{i}})+[S]}$$

(2) $$V=\frac{V_{max}[S]}{{(K}_{m}+[S])(1+\frac{\left[I\right]}{K_{i}})}$$

(3) $$V=\frac{V_{max}[S]}{K_{m}(1+\frac{\left[I\right]}{K_{i}})[S]}$$

(4) $$V=\frac{V_{max}[S]}{K_{m}\left(1+\frac{\left[I\right]}{K_{i}}\right)+[S]\left(1+\frac{\left[I\right]}{{\alpha K}_{i}}\right)}$$

where *V* and *V*_max_ are the reaction velocity and maximum reaction velocity, respectively; [*S*] and [*I*] are substrate and inhibitor concentrations; *K*_m_ is the Michaelis constant; *K*_i_ is the inhibition constant; and alpha indicates how the inhibitor affects the enzyme’s substrate affinity. Each inhibition model was fitted to the data to identify the most suitable inhibition type. Graphical representations of the inhibition type and *K*_i_ value were generated using Lineweaver–Burk [41] and Dixon plots [42].

### 4.5. Molecular Docking and Protein–Ligand Interaction Analysis

No complete experimentally determined crystal structures of human UGT1A1 or UGT1A9 with a fully resolved substrate-binding pocket are currently deposited in the RCSB Protein Data Bank. Accordingly, three-dimensional structures of UGT1A1 (UniProt P22309) and UGT1A9 (UniProt O60656) were retrieved from the AlphaFold Protein Structure Database (https://alphafold.ebi.ac.uk) [19]. Models with predicted local distance difference test (pLDDT) scores > 90 across the catalytic domain were selected to ensure structural accuracy in the substrate-binding region. The reliability of AlphaFold2 models for virtual screening and molecular docking has been independently validated [43,44], and the consistency between our docking results and established UGT catalytic mechanisms further supports the structural approach. Each model was energy-minimised and refined around the catalytic pocket prior to docking. Protein structures were prepared using AutoDock Tools 1.5.6 by removing water molecules, adding polar hydrogens, and assigning Gasteiger partial charges. Ligand structures of fraxetin, fraxin, esculetin, esculin, and daphnetin were obtained from PubChem (https://pubchem.ncbi.nlm.nih.gov, accessed on 1 February 2026) and energy-minimised prior to docking. Molecular docking was performed using AutoDock Vina 1.2.0 [45], with search grids centred on the catalytic pocket. The lowest-energy binding pose was selected for each compound–enzyme pair for further analysis. Protein–ligand interaction profiling was conducted using the Protein–Ligand Interaction Profiler (PLIP) online server (https://plip-tool.biotec.tu-dresden.de, accessed on 10 February 2026) to characterise hydrogen bonds, hydrophobic contacts, salt bridges, and π-stacking interactions [46]. Binding free energies (Δ*G*, kcal/mol) are reported as the lowest docked energy of the optimal binding pose. To validate that the docking protocol identifies the catalytically relevant substrate-binding site rather than an unrelated cavity, two established clinical UGT inhibitors were benchmark-docked into their respective AlphaFold-derived models under the same search-grid parameters used for the test compounds. Atazanavir, a clinical UGT1A1 inhibitor known to elevate unconjugated bilirubin via inhibition of UGT1A1-mediated glucuronidation, was docked into UGT1A1 and engaged 8 of the 11 fraxetin pocket residues (73% overlap), including 4 of the 5 canonical UGT1A1 catalytic-network residues (His39, His372, Ser375, Asp396), with the top-ranked contact His39 at 2.86 Å; the ligand pose centroid was located 8.7 Å from the fraxetin pocket CA centroid, confirming convergence to the catalytic substrate site. Niflumic acid, a selective UGT1A9 inhibitor widely used as a positive control in UGT inhibition screening, was docked into UGT1A9 and engaged 3 of the 5 catalytic-network residues (Ser306 at 3.05 Å, His373 at 3.68 Å, Glu377 at 3.39 Å) together with adjacent substrate-binding pocket residues (Ser303, Gly305), with a pose centroid 5.8 Å from the catalytic-site centroid. These benchmarks demonstrate that the docking protocol reliably places known UGT inhibitors at the catalytic substrate-binding site of both isoforms, supporting the validity of the fraxetin pose predictions (Supplementary Table S5).

### 4.6. Molecular Dynamics Simulations and MM/GBSA/PBSA Analysis

Molecular dynamics (MD) simulations were performed using GROMACS 2022 [47] with the AMBER14SB force field for the protein and GAFF2 for the ligand [48]. Ligand topology files were generated using sobtop 1.0 (dev3.1) with RESP charge assignment. The TIP3P water model was used for solvation in a cubic box with 1.0 nm padding from the protein surface. The system was neutralised by adding Na^+^ and Cl^−^ ions at a physiological concentration of 0.15 M NaCl using the gmx genion tool. Energy minimization was performed in three sequential stages: first with restraints on the solute, then with restraints on counterions, and finally without restraints on the full system. Each stage employed the steepest descent algorithm (3000 steps) followed by the conjugate gradient algorithm (2000 steps). Long-range electrostatic interactions were treated using the particle mesh Ewald (PME) method with a 1.0 nm cutoff. Bond constraints were handled using the LINCS algorithm with a 2 fs integration time step. Production simulations were run for 100 ns under NPT ensemble conditions (310 K, 1 bar) using the Nosé-Hoover thermostat and Parrinello-Rahman barostat. Structural stability and dynamics were evaluated using the following GROMACS analysis tools: root mean square deviation (RMSD; gmx rms), root mean square fluctuation (RMSF; gmx rmsf), radius of gyration (Rg; gmx gyrate), solvent-accessible surface area (SASA; gmx sasa), and hydrogen bond count (gmx hbond). Ligand binding stability was additionally assessed independently of protein stability by computing the ligand heavy-atom RMSD relative to the docking-derived starting pose (after least-squares superposition on protein Cα atoms) and the degree of ligand burial (the fraction of ligand heavy atoms with at least one protein heavy atom within 4 Å). Free energy landscape (FEL) analysis was performed using principal component analysis (PCA); the first two principal components (PC1 and PC2) were used as reaction coordinates, and the Gibbs free energy landscape was constructed using gmx covar, gmx anaeig, and gmx sham. Both MM/GBSA and MM/PBSA binding free energies were computed with the gmx_MMPBSA package [49] on evenly spaced frames sampled across the production trajectory of each replicate; the reported binding free energies are therefore ensemble averages over the sampled bound conformations rather than single-pose interaction energies. Per-residue energy decomposition was examined but, owing to inter-replicate variability comparable to the mean for the principal contributors, is not reported quantitatively. 

### 4.7. In Vitro–In Vivo Extrapolation and DDI Risk Assessment

In vivo herb-drug interaction potential was predicted using the basic static model recommended by the FDA 2020 DDI Guidance for reversible inhibition [4]. The systemic inhibition risk ratio (*R*_1_) and intestinal inhibition risk ratio (*R*_1,gut_) were calculated as

$$R_{1}=1+\frac{f_{u,p}\times C_{max}}{K_{i}}$$

$$R_{1,gut}=1+\frac{I_{gut}}{K_{i}}$$

where *C*_max_ is the maximum plasma concentration of the test compound, *f*_u,p_ is the unbound fraction in plasma, and *I*_gut_ is the estimated intestinal inhibitor concentration calculated as dose/250 mL. The inhibition constant *K*_i_ derived from the Lineweaver–Burk slope replot was used, consistent with FDA guidance recommendations for reversible inhibitors. Per FDA guidance, *R*_1_ ≥ 1.02 indicates potential for clinically significant systemic HDI, and *R*_1,gut_ ≥ 11 indicates high potential for intestinal DDI. Pharmacokinetic parameters were derived from published preclinical studies, and multiple *C*_max_ scenarios were evaluated to capture the range of exposure conditions reported across species and dose levels. In the absence of experimentally measured plasma protein binding data, a conservative worst-case assumption of *f*_u,p_ = 1.0 was adopted, as recommended for initial DDI risk screening [4]. Sensitivity analyses were conducted across multiple *C*_max_ and *f*_u,p_ scenarios to assess the robustness of the risk predictions.

## 5. Conclusions

Fraxetin is a moderate-potency mixed-type inhibitor of UGT1A1 (IC_50_ = 15.99 μM, *K*_i_ = 8.32 μM, *α* = 3.77) and UGT1A9 (IC_50_ = 8.44 μM, *K*_i_ = 5.90 μM, *α* = 1.89), with a modest (~1.4-fold, *K*_i_-based) preference toward UGT1A9. Structure–activity analysis delineated a dual-element pharmacophore—the C-6 methoxy group and the 7,8-dihydroxycoumarin aglycone—both required for potent dual-isoform inhibition. Computational analysis (molecular docking, PLIP, and triplicate MD with MM/GBSA) provided directionally concordant, ensemble-level support for the UGT1A9 preference, while indicating that the docking-derived pose was not retained during simulation and that the selectivity should not be ascribed to a specific residue-level interaction. FDA 2020 IVIVE analysis identified the intestinal compartment as the primary site of potentially clinically meaningful inhibition, with *R*_1,gut_ approximately five- to seven-fold above the regulatory threshold of 11 (luminal concentration capped at fraxetin solubility); systemic risk was borderline and conditional on worst-case exposure assumptions (*R*_1_ = 1.21–2.83). On this basis, intestinal UGT1A1 and UGT1A9 substrates of narrow therapeutic index, such as irinotecan and dapagliflozin, are the priority for clinical follow-up. These findings warrant clinical vigilance when Cortex Fraxini-containing preparations are co-administered with narrow-therapeutic-index drugs primarily cleared by these isoforms, pending confirmation by PBPK modelling and clinical interaction studies. Accordingly, the present results should be interpreted as a potential interaction signal requiring prospective confirmation rather than as an established clinical risk.

## Figures and Tables

**Table 1:** Inhibition kinetic parameters of fraxetin toward UGT1A1 and UGT1A9.

Enzyme	IC_50_ (μM)	*K*_i_ (μM)	*α*	Inhibition Type
*UGT1A1*	15.99 ^a^ (13.76–18.65)	8.32	3.77	Mixed (preferentially competitive)
*UGT1A9*	8.44 ^b^ (7.36–9.69)	5.90	1.89	Mixed (preferentially competitive)

^a,b^ 95% confidence intervals shown in parentheses. *K*_i_, inhibition constant; *α*, factor by which *K*_i_ is modified upon inhibitor binding to the enzyme–substrate complex (*α* > 1 indicates preferentially competitive character of mixed-type inhibition).

**Table 2:** Molecular docking, binding free energies and in vitro inhibitory activity of fraxetin and structural analogues against UGT1A1 and UGT1A9.

Compound	Structural Feature	Δ*G* UGT1A1 (kcal/mol)	Δ*G* UGT1A9 (kcal/mol)	In Vitro Activity (100 μM)
*Fraxetin*	7,8-OH; C-6-OCH_3_; aglycone	−6.8	−7.6	>80% inhibition (UGT1A1 and UGT1A9)
*Fraxin*	7,8-OH; C-6-OCH_3_; 8-O-glucoside	−8.2	−8.9	UGT1A1 ~68%; UGT1A9 ~10%
*Daphnetin*	7,8-OH; no OCH_3_; aglycone	−6.3	−7.2	UGT1A1 ~47%; UGT1A9 ~58%

Δ*G*, binding free energy calculated by AutoDock Vina 1.2.0. In vitro inhibitory activity determined at 100 μM using 4-MU as a probe substrate.

**Table 3:** IVIVE-predicted herb–drug interaction risk ratios for fraxetin toward UGT1A1 and UGT1A9.

Scenario	*C*_max_ (μM)	*f*_u,p_	[*I*]_max,u_ (μM)	*R*_1_ UGT1A1	*R*_1_ UGT1A9	*R*_1,gut_ UGT1A1	*R*_1,gut_ UGT1A9
Rat (fraxin, 50 mg/kg)	10.80	1.0	10.80	2.30 ↑	2.83 ↑	53 ↑↑	74 ↑↑
Rat (fraxetin, 25 mg/kg)	2.40	1.0	2.40	1.29 ↑	1.41 ↑	53 ↑↑	74 ↑↑
Beagle dog	1.78	1.0	1.78	1.21 ↑	1.30 ↑	53 ↑↑	74 ↑↑

*R*_1_ = 1 + [*I*]_max,u_/*K*_i_; *R*_1,gut_ = 1 + *I*_gut_/*K*_i_; *I*_gut_ = Dose/250 mL, capped at the aqueous solubility limit (≈0.43 mM). For an illustrative oral dose of 500 mg fraxetin equivalent (MW 208.17 g/mol), the uncapped nominal *I*_gut_ would be ≈9608 μM; as this exceeds the solubility limit, the attainable *I*_gut_ is capped at ≈0.43 mM, from which the tabulated *R*_1,gut_ values are calculated. *K*_i_, inhibition constant from mixed-type inhibition kinetics; *f*_u,p_ = 1.0, worst-case assumption. ↑, exceeds *R*_1_ ≥ 1.02 threshold; ↑↑, exceeds *R*_1,gut_ ≥ 11 threshold.

**Table 4:** Contrast between current knowledge of the UGT-inhibitory potential of Cortex Fraxini coumarins and the contribution of the present study.

Aspect	Current Knowledge (Prior Literature)	This Study
UGT inhibition screening	Not systematically assessed; only isolated reports on non-UGT targets	First systematic screen of five coumarins against eleven human UGT isoforms; UGT1A1 and UGT1A9 identified as primary targets
Inhibition mechanism and potency	Not reported	Mixed-type (mainly competitive) inhibition; *K*_i_ 8.32 μM (UGT1A1) and 5.90 μM (UGT1A9); moderate potency
Structural basis of binding	No binding model	Binding localised to the UGT aglycone-acceptor site; hydrogen bonding of the 7,8-dihydroxy and 6-methoxy groups rationalises the modest (~1.4-fold) UGT1A9-over-UGT1A1 preference
Clinical-risk translation	No exposure-based assessment	Exposure-anchored, solubility-constrained risk: robust in the intestinal lumen (*R*_1,gut_ ≈ 53–74, ~5–7-fold above threshold) but borderline systemically (*R*_1_ ≈ 1.2–2.8); a compartment-resolved hazard signal

**Table 5:** The five coumarin constituents of Cortex Fraxini examined in this study, their approximate reported content, and the rationale for their selection.

Constituent	Chemical Class	Content (% Dry wt)	Form	Rationale for Inclusion
Esculin	Coumarin 6-O-glucoside	2–8	Glycoside (prodrug)	Most abundant constituent; intestinal source of esculetin
Fraxin	Coumarin 8-O-glucoside	2–5	Glycoside (prodrug)	Abundant constituent; intestinal source of fraxetin
Fraxetin	Dihydroxy-methoxycoumarin aglycone	0.1–0.5 (plus from fraxin)	Aglycone	Predominant circulating species; primary exposure-relevant inhibitor
Esculetin	6,7-Dihydroxycoumarin aglycone	Minor (from esculin)	Aglycone	Aglycone of esculin; structural comparator
Daphnetin	7,8-Dihydroxycoumarin aglycone	Trace/minor	Aglycone	Positional isomer of esculetin; substitution-pattern comparator

## Data Availability

The original contributions presented in this study are included in the article/Supplementary Material. Further inquiries can be directed to the corresponding authors.

## Supplementary Materials

The following supporting information can be downloaded at: https://www.mdpi.com/article/10.3390/ph19060968/s1, Table S1. Residual UGT activity and statistical significance for the five Cortex Fraxini constituents across 11 recombinant human UGT isoforms; Table S2. Protein–ligand interactions identified by PLIP v2.4.0 analysis for fraxetin, fraxin, and daphnetin in complex with UGT1A1 and UGT1A9; Table S3. Inhibitory potency of fraxetin in the context of representative UGT1A1 and UGT1A9 inhibitors; Table S4. Predicted herb–drug interaction risk for major UGT1A1 and UGT1A9 substrate drugs co-administered with fraxetin-containing Cortex Fraxini preparations; Table S5. Detailed benchmark-docking contact residues for atazanavir (UGT1A1) and niflumic acid (UGT1A9); Table S6. Replicate-level IC_50_ and inhibition kinetic parameters of fraxetin against UGT1A1 and UGT1A9; Table S7. Sensitivity analysis of IVIVE-predicted systemic risk ratio (*R*_1_) across three *C*_max_ exposure scenarios and three *f*_u,p_ assumptions.

## Abbreviations

The following abbreviations are used in this manuscript:

UGT	UDP-glucuronosyltransferase
HDI	Herb–drug interaction
DDI	Drug–drug interaction
TCM	Traditional Chinese medicine
4-MU	4-Methylumbelliferone
HPLC	High-performance liquid chromatography
IVIVE	In vitro–in vivo extrapolation
PLIP	Protein–Ligand Interaction Profiler
MD	Molecular dynamics
MM/PBSA	Molecular mechanics/Poisson–Boltzmann surface area
RMSD	Root mean square deviation
RMSF	Root mean square fluctuation
SASA	Solvent-accessible surface area
FEL	Free energy landscape
PCA	Principal component analysis
FDA	Food and Drug Administration
DMSO	Dimethyl sulfoxide
UDPGA	Uridine 5′-diphosphoglucuronic acid
