# Supplementary material for: Fraxetin Inhibits UGT1A1 and UGT1A9 Activities In Vitro: Inhibition Kinetics, Molecular Dynamics Simulation, and Prediction of Herb–Drug Interaction Risk
Source: Pharmaceuticals (Basel). 2026 Jun 22;19(6):968. doi: 10.3390/ph19060968 (PMC13304715; doi:10.3390/ph19060968)
Supplement: Supplementary file 1 [file pharmaceuticals-19-00968-s001.zip › pharmaceuticals-4360995-supplementary.pdf]

Supplementary Materials

Fraxetin Inhibits UGT1A1 and UGT1A9 Activities In Vitro: Inhibition Kinetics, Molecular Dynamics Simulation, and Prediction of Herb–Drug Interaction Risk

Jinqian Chen, Han Han, Jibin Li, Simeng Xu, Xichuan Li*, Zhenyu Zhao*

Manuscript ID: pharmaceuticals-4289911

Contents

Table S1. Residual UGT activity and statistical significance for the five Cortex Fraxini constituents across 11 recombinant human UGT isoforms.

Table S2. Protein–ligand interactions identified by PLIP v2.4.0 analysis for fraxetin, fraxin, and daphnetin in complex with UGT1A1 and UGT1A9.

Table S3. Inhibitory potency of fraxetin in the context of representative UGT1A1 and UGT1A9 inhibitors.

Table S4. Predicted herb–drug interaction risk for major UGT1A1 and UGT1A9 substrate drugs co-administered with fraxetin-containing Cortex Fraxini preparations.

Table S5. Detailed benchmark-docking contact residues for atazanavir (UGT1A1) and niflumic acid (UGT1A9).

Table S6. Replicate-level IC_{50} and inhibition kinetic parameters of fraxetin against UGT1A1 and UGT1A9.

Table S7. Sensitivity analysis of IVIVE-predicted systemic risk ratio (R_1) across three C_{max} exposure scenarios and three $f_{u,p}$ assumptions.

Table S1. Residual UGT activity and statistical significance for the five Cortex Fraxini constituents across 11 recombinant human UGT isoforms. Each constituent (100 μM) was compared with the vehicle control by two-way ANOVA (isoform × compound) followed by Dunnett's multiple-comparison test (within each isoform; one family per row; multiplicity-adjusted P values). Residual activity is the mean of triplicate determinations (vehicle control = 100%).

UGT isoform	Constituent	Residual activity (%)	Mean difference vs. control	95% CI of difference	Adjusted P value	Significance
**UGT1A1**	Fraxetin	16.4	83.54	76.91 to 90.18	<0.0001	****
	Fraxin	31.9	68.14	61.51 to 74.78	<0.0001	****
	Esculetin	55.6	44.44	37.81 to 51.07	<0.0001	****
	Esculin	69.6	30.38	23.74 to 37.01	<0.0001	****
	Daphnetin	52.6	47.43	40.79 to 54.06	<0.0001	****
**UGT1A3**	Fraxetin	39.5	60.51	53.87 to 67.14	<0.0001	****
	Fraxin	46.9	53.10	46.47 to 59.74	<0.0001	****
	Esculetin	76.7	23.35	16.72 to 29.99	<0.0001	****
	Esculin	84.1	15.92	9.285 to 22.55	<0.0001	****
	Daphnetin	83.1	16.89	10.26 to 23.52	<0.0001	****
**UGT1A6**	Fraxetin	39.9	60.07	53.43 to 66.70	<0.0001	****
	Fraxin	26.8	73.16	66.53 to 79.79	<0.0001	****
	Esculetin	57.5	42.55	35.92 to 49.19	<0.0001	****
	Esculin	77.0	22.97	16.33 to 29.60	<0.0001	****
	Daphnetin	38.0	61.96	55.33 to 68.59	<0.0001	****
**UGT1A7**	Fraxetin	31.8	68.19	61.55 to 74.82	<0.0001	****
	Fraxin	51.7	48.26	41.63 to 54.90	<0.0001	****
	Esculetin	66.4	33.56	26.93 to 40.20	<0.0001	****
	Esculin	76.6	23.37	16.74 to 30.01	<0.0001	****
	Daphnetin	52.6	47.39	40.75 to 54.02	<0.0001	****
**UGT1A8**	Fraxetin	96.3	3.69	-2.939 to 10.33	0.4795	ns

	Fraxin	83.5	16.49	9.855 to 23.12	<0.0001	****
	Esculetin	87.0	13.04	6.405 to 19.67	<0.0001	****
	Esculin	97.3	2.66	-3.975 to 9.289	0.7615	ns
	Daphnetin	93.5	6.48	-0.1555 to 13.11	0.0581	ns
**UGT1A9**	Fraxetin	8.0	92.04	85.41 to 98.67	<0.0001	****
	Fraxin	90.0	9.99	3.361 to 16.63	0.0009	***
	Esculetin	58.3	41.74	35.11 to 48.37	<0.0001	****
	Esculin	87.9	12.07	5.441 to 18.71	<0.0001	****
	Daphnetin	42.4	57.63	51.00 to 64.26	<0.0001	****
**UGT1A10**	Fraxetin	87.4	12.58	5.951 to 19.22	<0.0001	****
	Fraxin	87.7	12.31	5.681 to 18.95	<0.0001	****
	Esculetin	51.1	48.86	42.23 to 55.49	<0.0001	****
	Esculin	80.2	19.77	13.13 to 26.40	<0.0001	****
	Daphnetin	50.8	49.23	42.60 to 55.86	<0.0001	****
**UGT2B4**	Fraxetin	88.0	11.98	5.348 to 18.61	<0.0001	****
	Fraxin	94.2	5.81	-0.8188 to 12.45	0.1063	ns
	Esculetin	87.4	12.60	5.971 to 19.24	<0.0001	****
	Esculin	83.9	16.07	9.441 to 22.71	<0.0001	****
	Daphnetin	70.0	30.05	23.41 to 36.68	<0.0001	****
**UGT2B7**	Fraxetin	72.0	27.95	21.32 to 34.59	<0.0001	****
	Fraxin	63.4	36.62	29.98 to 43.25	<0.0001	****
	Esculetin	77.9	22.13	15.50 to 28.76	<0.0001	****
	Esculin	95.3	4.73	-1.905 to 11.36	0.2502	ns
	Daphnetin	76.6	23.42	16.78 to 30.05	<0.0001	****
**UGT2B15**	Fraxetin	84.7	15.30	8.668 to 21.93	<0.0001	****
	Fraxin	81.3	18.67	12.03 to 25.30	<0.0001	****

	Esculetin	90.3	9.69	3.058 to 16.32	0.0014	**
	Esculin	96.9	3.08	-3.549 to 9.715	0.6461	ns
	Daphnetin	91.3	8.65	2.015 to 15.28	0.0054	**
**UGT2B17**	Fraxetin	97.5	2.50	-4.132 to 9.132	0.8007	ns
	Fraxin	95.2	4.85	-1.785 to 11.48	0.2295	ns
	Esculetin	100.1	-0.14	-6.775 to 6.489	>0.9999	ns
	Esculin	96.2	3.84	-2.795 to 10.47	0.4426	ns
	Daphnetin	95.9	4.11	-2.519 to 10.75	0.3758	ns

Notes: Significance levels: ****, $P < 0.0001$; ***, $P < 0.001$; **, $P < 0.01$; *, $P < 0.05$; ns, not significant ($P \geq 0.05$). Two-way ANOVA: compound, $F(5,132) = 683.0$, $P < 0.0001$; isoform, $F(10,132) = 402.4$, $P < 0.0001$; interaction, $F(50,132) = 72.26$, $P < 0.0001$.

Table S2. Protein–ligand interactions identified by PLIP v2.4.0 analysis for fraxetin, fraxin, and daphnetin in complex with UGT1A1 and UGT1A9.

**Interaction type**	**Residue**	**Ligand atom/group**	**Distance (Å)**	**Notes**
UGT1A1–Fraxetin (ΔG = −6.8 kcal/mol; >80% inhibition)				
Hydrophobic	PHE394	C-6-OCH_3 (C)	3.80	Hydrophobic anchor
Hydrophobic	PHE394	C-6-OCH_3 (C)	3.94	Hydrophobic anchor
Hydrogen bond	SER309	O2 (catechol C-7)	2.91	Protein donor
Hydrogen bond	MET310	O2 (catechol C-7)	3.15	Protein donor
Hydrogen bond	SER375	O3 (catechol C-8)	3.30	Protein donor
Hydrogen bond	HIS376	O3 (catechol C-8)	3.04	Protein donor
Salt bridge	HIS372	O2/O3	4.51	Catalytic His
T-π stacking	HIS372	Coumarin ring	4.62	Angle 64.91°; catalytic His
UGT1A9–Fraxetin (ΔG = −7.6 kcal/mol; >80% inhibition)				
Hydrophobic	PHE391	C-6-OCH_3 (C)	3.95	Hydrophobic anchor
Hydrogen bond	SER306	O2 (catechol C-7)	2.88	Protein donor

Hydrogen bond	MET307	O2 (catechol C-7)	3.04	Protein donor
Hydrogen bond	SER372	O3 (catechol C-8)	3.14	Protein donor
Hydrogen bond	HIS373	O3 (catechol C-8)	2.99	Protein donor (bidirectional)
Hydrogen bond	HIS373	O3 (catechol C-8)	3.24	Ligand donor → His N2 acceptor
Hydrogen bond	SER36	O3 (catechol C-8)	4.05	Weak/peripheral
Salt bridge	HIS369	O2/O3	4.61	Catalytic His
T-π stacking	HIS369	Coumarin ring	4.52	Angle 74.17°; catalytic His
UGT1A1–Fraxin (ΔG = −8.2 kcal/mol; UGT1A1 ~68% inhibition; UGT1A9 ~10% inhibition)				
Hydrogen bond	SER309	O3 (glucose OH)	2.99	Protein donor; glucose, not coumarin O2
Hydrogen bond	SER309	O3 (glucose OH)	2.84	Ligand donor → SER sidechain acceptor
Hydrogen bond	MET310	O3 (glucose OH)	3.06	Protein donor; glucose hydroxyl
Hydrogen bond	SER375	O3 (glucose OH)	3.06	Protein donor; glucose hydroxyl
Hydrogen bond	HIS376	O3 (glucose OH)	3.10	Protein donor; glucose hydroxyl
Hydrogen bond	SER38	O3 (glucose OH)	3.97	Peripheral; glucose hydroxyl
Hydrogen bond	GLY374	O3 (glucose OH)	3.87	Peripheral; ligand donor
Hydrogen bond	ASP396	O3 (glucose OH)	3.86	Peripheral; ligand donor
Hydrogen bond	GLN397	O3 (glucose OH)	3.83	Peripheral; glucose hydroxyl
Salt bridge	HIS372	O2/O3	4.75	Retained; coumarin

				nucleus displaced from catalytic site
T-π stacking	—	—	—	Absent (vs. fraxetin complex)
UGT1A9–Fraxin (ΔG = −8.9 kcal/mol; UGT1A9 ~10% inhibition; high-affinity but non-productive binding)				
Hydrogen bond	HIS278	O3 (glucose OH)	3.35	Peripheral; ligand donor
Hydrogen bond	LEU352	O2 (glucose OH)	3.05	Peripheral; protein donor
Hydrogen bond	GLN354	O3 (glucose OH)	3.59	Peripheral; protein donor
Hydrogen bond	HIS373	O3 (glucose OH)	3.97	Peripheral; ligand donor
Hydrogen bond	GLU377	O3 (glucose OH)	3.02	Peripheral; bidirectional
Parallel π-stacking	TRP351	Glucose ring	3.98	Angle 8.76°; UGT1A9-specific; displaces coumarin from catalytic site
Hydrophobic	—	—	—	Absent (PHE391 contact lost)
Salt bridge	—	—	—	Absent (HIS369 disengaged)
UGT1A1–Daphnetin (ΔG = −6.3 kcal/mol; UGT1A1 ~47% inhibition)				
Hydrogen bond	SER309	O2 (catechol C-7)	3.04	Protein donor; C-6-OCH_3 absent
Hydrogen bond	SER375	O3 (catechol C-8)	3.26	Protein donor
Hydrogen bond	HIS376	O3 (catechol C-8)	3.69	Protein donor
Salt bridge	HIS372	O2/O3	4.53	Retained
T-π stacking	HIS372	Coumarin ring	4.54	Angle 71.60°; retained
Hydrophobic	—	—	—	Absent (PHE394

				contact lost; no C-6-OCH_3)
Hydrogen bond	—	MET310	—	Absent (vs. fraxetin complex)
UGT1A9–Daphnetin (ΔG = −7.2 kcal/mol; UGT1A9 ~58% inhibition; complete binding-mode reorientation)				
Hydrophobic	ILE193	Coumarin C (ring)	3.63	Alternative anchor; remote from catalytic site
Hydrogen bond	PRO191	O3 (catechol C-8)	3.07	Ligand donor; peripheral
Hydrogen bond	PRO191	O3 (catechol C-8)	2.91	Ligand donor; peripheral
Hydrogen bond	ILE193	O3 (catechol C-8)	3.23	Protein donor; peripheral
Hydrogen bond	ARG400	O3 (catechol C-8)	3.69	Protein donor; peripheral
Salt bridge	ARG170	Carboxylate	4.26	Remote; dual ARG bridge
Salt bridge	ARG400	Carboxylate	3.56	Remote; dual ARG bridge
π-Cation	LYS263	Coumarin ring	5.47	Complete binding-mode collapse; PHE391/HIS369/Ser–Met–Ser–His all absent

Notes: Distances are donor–acceptor (D–A) distances for hydrogen bonds and centroid–centroid distances for π-stacking interactions. Complexes are grouped by section headers. —, interaction absent relative to the fraxetin complex. O2, hydroxyl oxygen at C-7; O3, hydroxyl oxygen at C-8 of the coumarin nucleus (fraxetin/daphnetin) or glucose moiety (fraxin). Receptor structures: UGT1A1 (UniProt P22309) and UGT1A9 (UniProt O60656), AlphaFold Monomer v2.0 predicted models retrieved from the AlphaFold Protein Structure Database (https://alphafold.ebi.ac.uk).

Table S3. Inhibitory potency of fraxetin in the context of representative UGT1A1 and UGT1A9 inhibitors.

Enzyme	Inhibitor	IC_{50} (μM)	K_i (μM)	Probe substrate	Potency class	Source

UGT1A1	Erlotinib / nilotinib / sorafenib	0.13–0.32	—	molidustat (HLM)	potent (clinical)	Mey et al. 2021
UGT1A1	Atazanavir (positive control)	0.4–2.3	1.9	β-estradiol / molidustat	potent (clinical)	Lv et al. 2019; Mey et al. 2021
UGT1A1	Silybin / 2,3-dehydrosilybin A	~2.1	—	bilirubin (HepG2)	moderate–potent (natural)	Vrba 2019
UGT1A1	Niflumic acid	22.2	14–18	β-estradiol / 4-MU	weak–moderate	Miners et al. 2011
UGT1A1	Fraxetin (this study)	16.0	8.3	4-MU	moderate	This study
UGT1A9	Niflumic acid (positive control)	0.034	0.028	4-MU	potent (selective)	Mano et al. 2006
UGT1A9	Diflunisal	1.31	0.71	4-MU	moderate–potent	Mano et al. 2006
UGT1A9	Ginsenoside Rc	6.4	2.8–3.3	4-MU / propofol	moderate (natural)	Lee 2019
UGT1A9	Norathyriol (mangiferin aglycone)	12.3	2.8	4-MU	moderate (natural)	Sun et al. 2017
UGT1A9	Diclofenac	24.2	53.3	4-MU	weak	Mano et al. 2006
UGT1A9	Fraxetin (this study)	8.4	5.9	4-MU	moderate	This study

Notes: Reported IC_{50}/K_i values are from the cited primary literature; probe substrate is indicated because absolute potency is substrate-dependent, and values obtained with 4-methylumbelliferone (4-MU) are most directly comparable to the present study. Fraxetin (K_i 8.3 μM for UGT1A1, 5.9 μM for UGT1A9) falls within the moderate-potency band: one to three orders of magnitude weaker than the prototypical potent inhibitors of each isoform (niflumic acid for UGT1A9, atazanavir/kinase inhibitors for UGT1A1), but comparable to dietary and herbal UGT inhibitors for which intestinal herb–drug interaction signals have been reported (norathyriol, ginsenoside Rc, milk-thistle flavonolignans). Source identifiers (PMID/PMC) are provided for reference assignment: PMID 16278927 (Mano et al., 4-MU NSAID panel, UGT1A9); PMID 21245288 (Miners et al., niflumic acid K_i); PMC6437557 (review; atazanavir K_i, HIV protease inhibitors); PMC7983974 (kinase inhibitors and atazanavir IC_{50}, molidustat HLM); Vrba 2019, Oxid. Med. Cell. Longev. (silybin/2,3-dehydrosilybin); Lee 2019 (ginsenoside Rc); PMC6152678 (norathyriol/mangiferin). Final reference numbers should be assigned in the main reference list.

Table S4. Predicted herb–drug interaction risk for major UGT1A1 and UGT1A9 substrate drugs upon co-administration with fraxetin-containing Cortex Fraxini preparations.

Substrate drug	UGT contribution	Therapeutic class	TI	Predicted ΔAUC	Clinical consequence of exposure increase
UGT1A1 substrate drugs					
Irinotecan (SN-38) [a]	UGT1A1 (~85%)	Antineoplastic	Narrow	21–130%↑	Severe diarrhoea, neutropenia, myelosuppression
Raltegravir	UGT1A1 (~70%)	HIV integrase inhibitor	Moderate	21–130%↑	Elevated drug exposure; CNS adverse effects
Atazanavir	UGT1A1 (substrate + inhibitor)	HIV protease inhibitor	Moderate	21–130%↑	Unconjugated hyperbilirubinaemia (overlap effect)
Etoposide	UGT1A1 (~40%)	Antineoplastic	Narrow	21–130%↑	Myelosuppression, mucositis
UGT1A9 substrate drugs					
Propofol	UGT1A9 (~70%)	Intravenous anaesthetic	Narrow	30–183%↑	Prolonged sedation, hypotension, respiratory depression
Mycophenolic acid	UGT1A9 (~50%) + UGT2B7	Immunosuppressant	Narrow	30–183%↑	Gastrointestinal toxicity, neutropenia, opportunistic infection
Sorafenib	UGT1A9 (major)	Multikinase inhibitor	Narrow	30–183%↑	Hand–foot skin reaction, hepatotoxicity, hypertension
Regorafenib	UGT1A9 (>50%)	Multikinase inhibitor	Narrow	30–183%↑	Hepatotoxicity, hand–foot skin reaction, fatigue
Diflunisal	UGT1A9 + UGT2B7	NSAID	Moderate	30–183%↑	Increased GI bleeding risk, renal impairment

Furosemide	UGT1A9	Loop diuretic	Moderate	30–183%↑	Electrolyte disturbance, dehydration

Notes: TI, therapeutic index. Predicted AUC fold-change (ΔAUC) was calculated from the FDA 2020 static-model risk ratio R_1 values determined in this study, using ΔAUC = (R_1 − 1) × 100%. R_1 range with $f_{u,p}$ = 1.0 (worst-case unbound): UGT1A1: 1.21–2.30 (21–130% ΔAUC); UGT1A9: 1.30–2.83 (30–183% ΔAUC). These predictions represent screening-level estimates per FDA DDI Guidance and require clinical pharmacokinetic verification. [a] Irinotecan is a prodrug activated by carboxylesterases to SN-38; UGT1A1 glucuronidates SN-38 to inactive SN-38G. UGT1A1 inhibition by fraxetin would impair SN-38 clearance, increasing toxic active metabolite exposure. Patients carrying UGT1A1*28 (homozygous Gilbert's syndrome phenotype) have baseline UGT1A1 activity reduced to ~30% of wild-type and would experience compounded risk. Luminal fraxetin exposure is solubility-constrained; the intestinal interaction risk is quantified separately in the main text.

Table S5. Detailed benchmark-docking contact residues for atazanavir (UGT1A1) and niflumic acid (UGT1A9). Protein residues within 4.5 Å heavy-atom distance of each docked ligand pose, ranked by minimum heavy-atom distance.

Rank	Residue	Min. distance (Å)	Role
(a) Atazanavir–UGT1A1 (top 20 of 25 total contacts)			
1	HIS39	2.86	fraxetin pocket; catalytic
2	GLN397	2.93	—
3	CYS177	3.20	—
4	HIS372	3.23	fraxetin pocket; catalytic
5	SER38	3.27	fraxetin pocket
6	LEU95	3.29	—
7	PRO176	3.34	—
8	GLY395	3.42	—
9	PRO196	3.46	—
10	LEU175	3.47	—
11	ASP396	3.50	fraxetin pocket; catalytic
12	PHE153	3.51	—
13	ALA174	3.51	—
14	SER375	3.51	fraxetin pocket; catalytic
15	ARG257	3.53	—

16	HIS173	3.55	—
17	ILE116	3.62	—
18	GLY374	3.65	fraxetin pocket
19	SER91	3.71	—
20	PHE92	3.78	—
(b) Niflumic acid–UGT1A9 (all 10 contacts)			
1	SER306	3.05	fraxetin pocket; catalytic
2	GLN354	3.10	—
3	LEU352	3.14	—
4	SER303	3.19	—
5	PHE39	3.31	—
6	GLU377	3.39	fraxetin pocket; catalytic
7	TRP351	3.45	—
8	GLY305	3.53	fraxetin pocket
9	HIS373	3.68	fraxetin pocket; catalytic
10	ARG333	3.70	—

Notes: Atazanavir achieved 8/11 (73%) overlap with fraxetin pocket residues and 4/5 engagement with the canonical UGT1A1 catalytic network (His39, His372, Ser375, Asp396), with His39 as the closest contact at 2.86 Å. Niflumic acid achieved 3/5 engagement with the UGT1A9 catalytic network (Ser306, His373, Glu377), with a pose centroid 5.8 Å from the catalytic-site centroid. These benchmark-docking results support the validity of the fraxetin pose predictions in both isoforms (see Methods Section 4.5 of main manuscript).

Table S6. Replicate-level IC_{50} and inhibition kinetic parameters of fraxetin against UGT1A1 and UGT1A9.

Parameter	Unit	Replicate 1	Replicate 2	Replicate 3	Mean ± SD [95% CI]
UGT1A1					
IC_{50}	μM	16.42	15.71	15.84	15.99 ± 0.38 [13.76–18.65]
K_{is} (slope replot)	μM	8.51	8.18	8.27	8.32 ± 0.17

K_{ii} (intercept replot)	μM	31.84	30.85	31.30	31.33 ± 0.50
α (K_{ii}/K_{is})	—	3.74	3.77	3.79	3.77 ± 0.02
R^2 (IC_{50} fit)	—	0.989	0.984	0.982	0.985 ± 0.004
R^2 (Lineweaver–Burk)	—	0.978	0.971	0.974	0.974 ± 0.003
UGT1A9					
IC_{50}	μM	8.61	8.32	8.39	8.44 ± 0.15 [7.36–9.69]
K_{is} (slope replot)	μM	5.96	5.84	5.90	5.90 ± 0.06
K_{ii} (intercept replot)	μM	11.24	11.02	11.16	11.14 ± 0.11
α (K_{ii}/K_{is})	—	1.89	1.89	1.89	1.89 ± 0.00
R^2 (IC_{50} fit)	—	0.994	0.991	0.992	0.992 ± 0.002
R^2 (Lineweaver–Burk)	—	0.987	0.982	0.985	0.985 ± 0.003

Notes: IC_{50} values were determined by nonlinear regression of dose–response curves (8 concentrations: 0, 1, 5, 10, 20, 40, 60, 80 μM; substrate 4-MU at the isoform-specific K_m) using GraphPad Prism 8.0; 95% confidence intervals are profile-likelihood derived from the global fit across the three replicates. K_{is} (slope replot) and K_{ii} (intercept replot) were determined by linear regression of Lineweaver–Burk secondary plots constructed independently for each replicate; $\alpha = K_{ii}/K_{is}$. Mixed-type inhibition was confirmed by the lowest Akaike Information Criterion (AIC) score compared to competitive, non-competitive, and uncompetitive models (ΔAIC > 8 for both isoforms). Inter-replicate coefficient of variation (CV%) was < 5% for all primary kinetic parameters.

Table S7. Sensitivity analysis of the FDA 2020 static-model–predicted systemic inhibition risk ratio (R_1) for fraxetin against UGT1A1 and UGT1A9 across three pharmacokinetic exposure scenarios and three plasma protein binding ($f_{u,p}$) assumptions.

PK scenario	C_{max} (μM)	$f_{u,p}$	$[I]_u$ (μM)	R_1 (UGT1A1)	R_1 (UGT1A9)	Risk flag
Beagle dog (low)	1.78	0.1	0.18	1.02	1.03	UGT1A1↑, UGT1A9↑
Beagle dog (low)	1.78	0.5	0.89	1.11	1.15	UGT1A1↑, UGT1A9↑
Beagle dog	1.78	1.0	1.78	1.21	1.30	UGT1A1↑,

(low)						UGT1A9↑
Rat (medium)	2.4	0.1	0.24	1.03	1.04	UGT1A1↑, UGT1A9↑
Rat (medium)	2.4	0.5	1.20	1.14	1.20	UGT1A1↑, UGT1A9↑
Rat (medium)	2.4	1.0	2.40	1.29	1.41	UGT1A1↑, UGT1A9↑
Rat (high)	10.8	0.1	1.08	1.13	1.18	UGT1A1↑, UGT1A9↑
Rat (high)	10.8	0.5	5.40	1.65	1.92	UGT1A1↑, UGT1A9↑
Rat (high)	10.8	1.0	10.80	2.30	2.83	UGT1A1↑, UGT1A9↑

Notes: $R_1 = 1 + (f_{u,p} \times C_{max})/K_i$, calculated per FDA 2020 DDI Guidance basic static model. K_i values: UGT1A1 K_{is} = 8.32 μM; UGT1A9 K_{is} = 5.90 μM (slope-derived from Lineweaver–Burk analysis). $[I]_u$ = unbound intracellular inhibitor concentration approximated as $f_{u,p} \times C_{max}$. $R_1 \geq 1.02$ indicates a screening-level flag warranting clinical investigation per FDA DDI Guidance. Both UGT1A1 and UGT1A9 risk flags were triggered in all 9 of 9 PK scenarios tested, including the most conservative case (beagle dog C_{max} 1.78 μM × $f_{u,p}$ 0.1). Cmax sources: beagle dog (fraxetin/Cortex Fraxini extract); rat medium (fraxetin 25 mg/kg p.o.); rat high (fraxin 50 mg/kg p.o. yielding fraxetin C_{max} via prodrug metabolism).
